# A Sensor for Detecting Aqueous Cu^2+^ That Functions in a Just-Add-Water Format

**DOI:** 10.1021/acsomega.4c08751

**Published:** 2024-12-19

**Authors:** Tyler J. Lucci, Abigail Neufarth, Jean-François Gaillard, Julius B. Lucks

**Affiliations:** † Department of Chemical and Biological Engineering, Northwestern University, 2145 Sheridan Road, Evanston, Illinois 60208, United States; ‡ Center for Synthetic Biology, Northwestern University, 633 Clark Street, Evanston, Illinois 60208, United States; § Center for Water Research, Northwestern University, 2205 Tech Drive, Evanston, Illinois 60208, United States; ∥ Department of Civil and Environmental Engineering, Northwestern University, 2145 Sheridan Road, Evanston, Illinois 60208, United States; ⊥ Interdisciplinary Biological Sciences Graduate Program, Northwestern University, 2205 Tech Drive, Evanston, Illinois 60208, United States; # Chemistry of Life Processes Institute, Northwestern University, 2170 Campus Drive, Evanston, Illinois 60208, United States

## Abstract

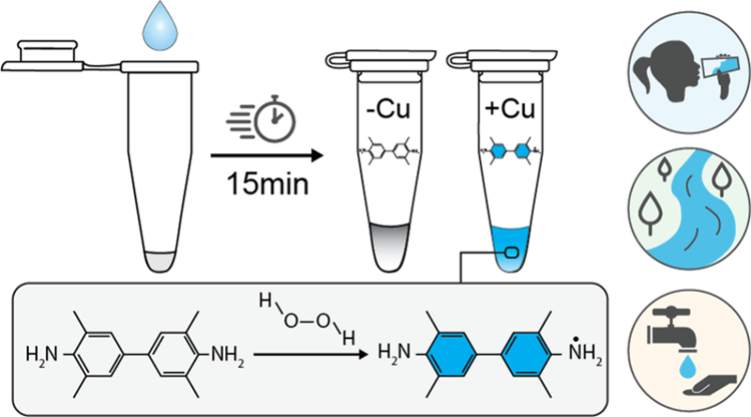

There is growing concern around the negative health impacts associated with contamination of drinking water by harmful chemicals. Technology that enables fast, cheap, and easy detection of ions and small molecules in drinking water is thus important for reducing the incidence of these negative health impacts. Here, we describe a sensor for detecting Cu^2+^ in water that provides colorimetric results in 15 min or less and functions in a just-add-water format. The sensor contains cheap reagents including salts, buffer, oxidant, chromogen, surfactant, and optionally a chelating agent. The sensor is assembled and lyophilized for shelf-stability and field-deployment. Rehydrating the sensor with water containing Cu^2+^ results in chromogen oxidation and blue color formation to visually indicate the presence of Cu^2+^. The sensor demonstrates high selectivity toward Cu^2+^ against other metal cations, functionality in field samples, shelf-stability, and can be tuned to activate at different Cu^2+^ threshold concentrations. This sensor thus has the potential to meet a variety of needs, such as point-of-need testing for Cu^2+^ to ensure water supplies meet health guidelines, such as the United States Environmental Protection Agency’s Lead and Copper Rule.

## Introduction

Reliable access to safe drinking water is a global challenge. Studies estimate that approximately one in four people lacks secure access to safe drinking water,^1^ while as much as 80% of the global population experiences high levels of threat to water security.^2^ In the United States, a major area of focus around drinking water quality is lead and copper contamination.^3^ As such, the United States Environmental Protection Agency (EPA) devised the Lead and Copper Rule (LCR), which specifies the permissible limits for lead and copper in drinking water.^3^ An ability to detect chemical contaminants in drinking water, such as lead and copper, is thus an important step toward improving water security.

In light of this, we sought to develop a point-of-need (PoN) sensor that will signal if a water sample contains copper at a concentration in excess of the US EPA’s Lead and Copper Rule limit of 1.3 ppm (20 μM).^3^ For user-friendliness, we aimed to develop a sensor that produces colorimetric results in 15 min or less and functions in a simple just-add-water format without requiring any ancillary equipment such as a blacklight or fluorimeter. We also aimed to develop a sensor that uses low-cost reagents and simple manufacturing so that it can be produced cheaply and distributed widely. In addition, we aimed for this sensor to only produce a colorimetric signal if the water contains copper at or in excess of 1.3 ppm, while avoiding color gradients that an end user would need to interpret. Such a sensor would allow an end user to rapidly test whether or not their drinking water is in compliance with the US EPA’s limit for copper in drinking water.

Fortunately, advances in synthetic biology and chemistry have enabled the development of PoN sensors for detecting many water contaminants, including copper.^4,5^ One approach for detecting water contaminants involves DNAzymes that cleave in the presence of a specific ligand.^5^ These cleaving DNAzymes are often deployed via a catalytic beacon approach, where the presence of the ligand, such as copper, drives cleavage, resulting in fluorophore-quencher separation and a detectable fluorescence signal.^5^

Although robust and promising, a typical limitation of fluorescence-based sensors is the requirement of ancillary equipment for readout, such as a blacklight or fluorimeter.^4,6,7^ To address this disadvantage, we initially sought to couple a colorimetric reporter system, known as the peroxidase-mimicking g-quadruplex DNAzyme,^8,9^ with cleaving DNAzymes^5^ to achieve one-pot colorimetric sensing of copper in water.

To investigate this sensing strategy, we chose a system involving a DNAzyme that cleaves in the presence of Cu^2+^ to yield a single-stranded oligo that would then function in the peroxidase-mimicking g-quadruplex DNAzyme reporter system, as has been studied previously.^10,11^ However, while experimenting, we identified reagent conditions (pH, buffer, salt) where the presence of Cu^2+^ alone resulted in a positive colorimetric signal, without requiring the peroxidase-mimicking g-quadruplex.

**Figure 1:**
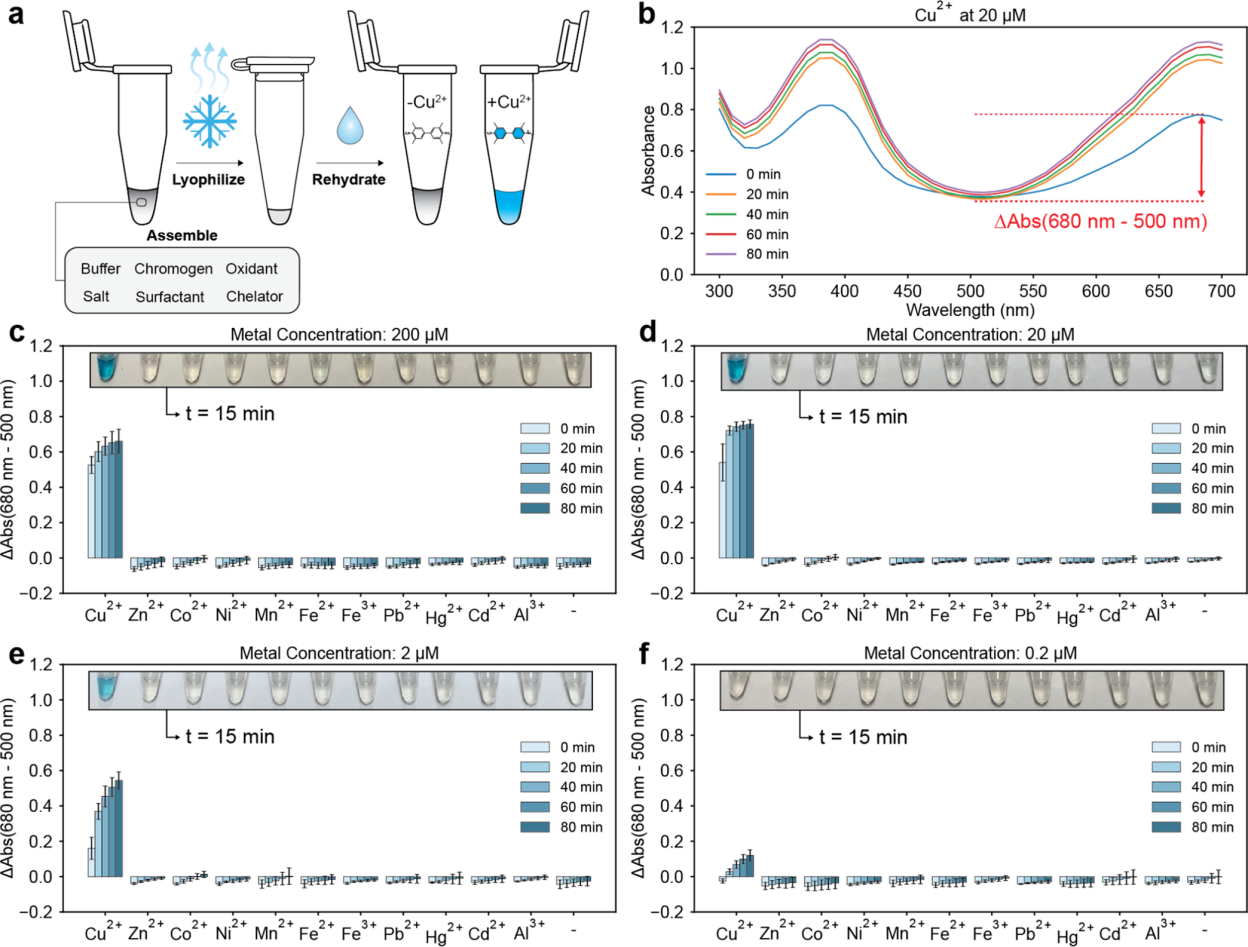
Sensor function and selectivity. Panel (a) presents the scheme for sensor assembly. Panel (b) presents the absorbance metric reported in panels (c–f), the difference in measured absorbance at 680 and 500 nm. Panels (c–f) present the selectivity assay examining the reactivity of the sensor at 25 °C toward metal cations in aqueous solution at the concentrations stated in the plot titles. “-“ indicates rehydration with 18.2 MΩ water. Upon rehydration, sensor components are 10 mM NaH_2_PO_4_, 9.4 mM NaOH, 2.5 mM SDS, 0.75 mM TMB, 8 mM H_2_O_2_, and 2.2 M NaCl. Photographs of sensor tubes are superimposed on each plot at 15 min after rehydration. Error bars are ± one standard deviation about the mean of three experimental replicates, each of which contained two technical replicates (*n* = 6).

By further refining our formulation, we developed a freeze-dried, field-deployable sensor for the colorimetric detection of Cu^2+^ in 15 min or less (Figure 1). Our initial formulation demonstrates naked eye detection of Cu^2+^ down to 2 μM with promising selectivity against other cations. Our subsequent formulation involves the use of a chelating agent, diethylenetriamine-pentaacetic acid penta-sodium salt (DTPA), to tune the sensor’s activation threshold so that it only produces visible color when Cu^2+^ concentration is near (within approximately 6 μM) or above the US EPA limit of 20 μM (1.3 ppm) for copper in drinking water. Finally, we demonstrate the sensor’s ability to function in field samples and evaluate its shelf stability.

## Experimental Section

Chemicals: The following were purchased from Sigma-Aldrich Co. (St. Louis, MO) and stored in sealed containers at room temperature: dimethyl sulfoxide (ACS Reagent SKU 472301–100 ML), sodium phosphate monobasic (ReagentPlus SKU S0751–100G), sodium chloride (ReagentPlus SKU 793566–2.5KG), sodium hydroxide (Reagent grade SKU S5881–1KG), sodium dodecyl sulfate (BioReagent SKU L3771–100G), ammonium sulfate (SKU A4418–500G), copper(II) chloride dihydrate (BioReagent SKU C3279–100G), zinc chloride (reagent grade SKU 208086–5G), cobalt(II) chloride hexahydrate (BioReagent SKU C8661–25G), nickel(II) chloride hexahydrate (ReagentPlus SKU 223387–25G), manganese(II) chloride tetrahydrate (BioReagent SKU M5005–100G), ammonium iron(II) sulfate hexahydrate (ACS Reagent SKU 215406–100G), iron(III) chloride hexahydrate (ACS Reagent SKU 236489–5G), lead(II) chloride (99.999% trace metals basis SKU 203572–10G), mercury chloride (ACS reagent SKU 215465–5G), cadmium(II) chloride (99.99% trace metals basis SKU 202908–10G), aluminum chloride hexahydrate (ReagentPlus SKU 237078-100G), magnesium chloride (anhydrous SKU M8266–1KG), calcium chloride (anhydrous, BioReagent SKU C5670–100G), sodium fluoride (ACS reagent SKU 201154–100G), iron(II) chloride tetrahydrate (ReagentPlus SKU 220299-5G), zinc sulfate heptahydrate (BioReagent SKU Z0251-100G), potassium chloride (SKU P9541-500G), sodium nitrate (ReagentPlus SKU S5506-250G), hydrochloric acid (BioReagent SKU H1758-500ML), sulfuric acid (ACS Reagent SKU 258105-500ML), sodium bisulfite (ACS Reagent SKU 243973-100G), and diethylenetriamine-pentaacetic acid penta-sodium salt solution (purum, ∼40% in H2O SKU 17969–500ML). 3,3′,5,5′-tetramethylbenzidine (SKU 860336–1G) was purchased from Sigma-Aldrich Co. (St. Louis, MO) and stored at 4 °C. Hydrogen peroxide 30% (ACS Reagent Cat. No. H1065) was purchased from Spectrum Chemical Mfg. Corp. (New Brunswick, NJ) and stored at 4 °C. Chlorox germicidal bleach concentrated (model no. S-19719) was purchased from Uline (Pleasant Prairie, WI). Hemin (Cat. No. HY-19424) was purchased from MedChemExpress (Monmouth Junction, NJ) and stored at −20 °C. Nucleic acid oligos were purchased from Integrated DNA Technologies (Coralville, IA) and stored at room temperature. 10× phosphate buffered saline (PBS) solution (Cat. No. BP399500) was purchased from Fisher Scientific (Hampton, NH) and stored at room temperature. Milli-Q water (18.2 MΩ) was dispensed via a Milli-Q system (Millipore, Billerica, MA).

### Stock Solutions

40 mM 3,3′,5,5′-tetramethylbenzidine stock solution was prepared by dissolving 9.6 mg 3,3′,5,5′-tetramethylbenzidine in 1 mL dimethyl sulfoxide, stored at −20 °C in 33 μL aliquots, and used within 2 months. 4 M sodium chloride stock solution was prepared by adding 2338 mg sodium chloride and raising the volume to 10 mL with 18.2 MΩ water, stored at room temperature, and used within 2 months. 1 M sodium hydroxide stock solution was prepared by adding 400 mg sodium hydroxide and raising the volume to 10 mL with 18.2 MΩ water, stored at room temperature, and used within 2 months. 0.1 M sodium dodecyl sulfate stock solution was prepared by dissolving 28.8 mg sodium dodecyl sulfate in 1 mL 18.2 MΩ water, stored at room temperature, and used within 2 months. 1 M sodium phosphate monobasic stock solution was prepared by adding 1200 mg sodium phosphate monobasic and raising the volume to 10 mL with 18.2 MΩ water, stored at room temperature, and used within 2 months. Metal stock solutions were prepared by dissolving solid metal salt in 18.2 MΩ water to yield 100 mM solution (except for lead chloride, which was made to 20 mM). The metal stock solutions were then serially diluted with 18.2 MΩ to lower concentrations. Metal solutions were made fresh weekly and stored in 1.5 mL snap-top tubes (Posi-Click Mfr. No. C2170).

### Sensor Assembly and Lyophilization

Sensor assembly involved preparing several stock solutions that were ultimately added to PCR tubes, which were then flash-frozen and lyophilized. Full sensor assembly instructions and reagent amounts, including tables and pictures, can be found in the Supporting Information including Figures S1–S3 and Supporting Flowsheets.

Each sensor was assembled in a PCR tube (Bio-Rad 0.2 mL Cat. No. TFI0201). In each PCR tube, 18 μL of solution (total) was added such that, upon rehydration with 20 μL of analyte solution after lyophilization, the final concentrations of the sensor components were: 10 mM sodium phosphate monobasic, 9.4 mM sodium hydroxide, 2.5 mM sodium dodecyl sulfate (SDS), 0.75 mM 3,3′,5,5′-tetramethylbenzidine (TMB), 8 mM hydrogen peroxide, and 2.2 M sodium chloride. Variable amounts of diethylenetriamine-pentaacetic acid penta-sodium salt solution (DTPA) were added to tune the threshold concentration of Cu^2+^ at which the sensor activates, as described in subsequent sections.

The general workflow for assembling the sensors was as follows. After adding buffer, surfactant, chromogen, salt, oxidant and optionally chelator, each PCR tube was flash-frozen on dry ice. Then, once all tubes were assembled, the tubes were further cooled in liquid nitrogen and then lyophilized. For lyophilization, a FreeZone 2.5 L −84 °C lyophilizer by Labconco was used at a set point of 0.04 mbar. The tubes were lyophilized for 16 h. Sensor tubes were either rehydrated immediately after lyophilization or packaged for storage before use (see the Shelf Life Study section).

### Absorbance Measurement

For absorbance measurement, the sensors were rehydrated inside their respective PCR tubes with 20 μL of analyte solution and then plated (20 μL) on a black 384-well optical bottom plate (Thermo Scientific Nunc Cat. No. 242764). The plate was then covered with a clear polyolefin seal (Thermo Scientific Cat. No. 232701). A photograph of the plate, backlit with a 359 lm light box (McMaster-Carr Cat. No. 1203T31), was then quickly taken with an IPhone XR on default photo settings with full zoom. A plate reader (Synergy H1 Microplate Reader by BioTek) was then used to measure the absorbance profile for each sensor. The program was specified to hold at 25 °C and measure absorbance spectra from 300 to 700 nm at 10 nm intervals every 15 min for a total of five measurements including the zero-time measurement. It is important to note that approximately 5 min is required to measure the absorbance spectra of 24 samples. Therefore, the interval between each absorbance spectrum measurement for all data provided is approximately 20 min. Immediately after absorbance measurement was finished, a photograph of the plate, backlit with the light box, was again taken with an IPhone XR on default photo settings with full zoom. For all analyses performed, sensors were rehydrated in a randomized fashion.

### Correlating Cu^2+^ with Absorbance at 450 nm

Sensors were rehydrated inside their respective PCR tubes with 20 μL of analyte solution and then incubated at 25 °C for 15 min on a thermocycler (Bio-Rad S1000 Thermal Cycler). 20 μL of 2 M sulfuric acid was then added to the sensors to stop the reactions. 20 μL of the stopped sensor reaction solution was then plated on a black 384-well optical bottom plate (Thermo Scientific Nunc Cat. No. 242764). The plate was then covered with a clear polyolefin seal (Thermo Scientific Cat. No. 232701). A photograph of the plate, backlit with a 359 lm light box (McMaster-Carr Cat. No. 1203T31), was then quickly taken with an IPhone XR on default photo settings with full zoom. A plate reader (Synergy H1 Microplate Reader by BioTek) was then used to measure the absorbance profile for each sensor. The program was specified to hold at 25 °C and measure absorbance spectra from 300 to 700 nm at 10 nm intervals.

### Sensor Photography

For photography of sensor tubes, the sensors were rehydrated inside their respective PCR tubes with 20 μL of analyte solution and then incubated (Labnet Vortemp 56) at 25 °C for 15 min before being photographed with an Iphone XR.

### Absorbance Data Analysis

The absorbance metric reported here, denoted ΔAbs (680–500 nm), was computed by subtracting the absorbance measured at 500 nm from the absorbance measured at 680 nm. This metric is presented throughout to infer the extent of sensor response, as we identified strong correlation between blue color formation and ΔAbs (680–500 nm) (Figures 1a and S8).

### Field Sample Testing

Field samples, which included municipal and environmental water samples, were gathered inside 15 mL polystyrene centrifuge tubes (Corning product number 352095). For testing, sensors were rehydrated with field samples and absorbance was measured as described in the Absorbance Measurement section. No pretreatment of the field samples was performed for testing with the sensor. For field samples with Cu^2+^ added, 2000 μM CuCl_2_ in 18.2 MΩ water was added to the field samples immediately before testing at 1:99 volume:volume to yield an added equivalent addition of 20 μM Cu^2+^. Field samples were sourced as follows with photographs taken by the authors: (1) Lake Michigan, Evanston, IL (environmental sample) (2) water dispenser, Evanston, IL (municipal sample) (3,8) sink sampled on two separate days, Evanston, IL (municipal samples) (4) sink, Evanston, IL (municipal sample) (5) sink, Evanston, IL (municipal sample) (6) sink, Orchard Park, NY, (municipal sample) (7) 18 Mile Creek, Derby, NY (environmental sample) (9) sink, Evanston, IL (municipal sample).

### FAAS Analysis

For FAAS analysis, field samples were pretreated by acidifying to pH ∼1 with ultrapure HNO_3_ (Fisher Chemical, catalog no. CAS7697–37–2) and concentrations were determined using a PerkinElmer PinAAcle 500 instrument, selecting the 324.8 nm wavelength for copper. The calibration was performed using appropriate dilutions of a Spex CertiPrep Cu standard—1000 mg/L—that ranged between 0.5 and 5 ppm by 0.5 ppm for copper, with a lower limit of quantitation about 20 ppb. Three replicates were performed and averaged, and error propagations were taken into account to estimate uncertainties (Figure S9).

### Shelf Life Study

Eleven sets of sensor tubes (one set for each day after day 0) were placed inside plastic packaging (O2frepak 2 Pack (Total 100Feet) 8 × 50 Rolls Vacuum Sealer Bags Rolls with BPA Free, Heavy Duty Vacuum Food Sealer Storage Bags Rolls, Cut to Size Roll, Great for Sous Vide, Amazon, ASIN B07BQP7733) with a desiccant card (Dri-Card Desiccants, Uline, catalog no. S-19582) and immediately vacuum sealed (KOIOS Vacuum Sealer Machine, 85Kpa Automatic Food Sealer, Amazon, ASIN B07FM3J6JF) following lyophilization. Each set of sensor tubes contained two tubes from each of three separate experimental batches plus one tube from a fourth experimental batch, yielding seven tubes total per set. Packaged sets of sensor tubes were stored at room temperature in a dark cabinet. The morning of each day, one set of sensor tubes was rehydrated with either 20 μM CuCl_2_ solution or 18.2 MΩ water (control) and the absorbances were measured as outlined in the Absorbance Measurement section. 20 μM CuCl_2_ solution was made fresh on day 0 and day 7. One tube was also rehydrated daily, incubated (Labnet Vortemp 56) at 25 °C, and photographed 15 min after rehydration with an IPhone XR.

### Minimum Components Study

Sensor reactions were assembled at room temperature. If present, the components were present at final concentrations of 10 mM NaH_2_PO_4_, 9.4 mM NaOH, 2.5 mM SDS, 0.75 mM TMB, and 8 mM H_2_O_2_. Full details on assembly can be found in the Supporting Flowsheets. CuCl_2_ was added to the reactions to yield a final concentration of 20 μM if copper was present, otherwise, 18.2 MΩ water was added to each reaction as a control. Once assembled, the reactions were plated and absorbance measured as described in the Absorbance Measurement section (incubation at 25 °C with spectra measured every 15 min).

## Results and Discussion

### Sensor Development

To develop a sensor for detecting copper in water with colorimetric readout, we initially sought to couple a Cu^2+^ dependent DNAzyme^6^ with the peroxidase mimicking g-quadruplex DNAzyme.^8^ In this system, the Cu^2+^ dependent DNAzyme would cleave its substrate strand to yield a single-stranded DNA oligo with repeating g-triplicates that could then complex with hemin to form the peroxidase-mimicking g-quadruplex DNAzyme and drive the oxidation of TMB in the presence of hydrogen peroxide to yield a colorimetric output^10^ (Figure S4).

Our vision was to achieve a sensor that functioned in a single tube format. Therefore, our initial experimentation involved assembling single tubes containing all the required components for the sensing reaction including the Cu^2+^ dependent DNAzyme, hemin, hydrogen peroxide, TMB, salts, and buffer. Fortunately, we noticed that the sensors with Cu^2+^ added turned blue. However, we also noticed that control sensors without hemin or DNA added also turned blue, but only when Cu^2+^ was added (Figure S5). Upon further development, we identified a sensor formulation that retained function after lyophilization and demonstrated good selectivity against other metal cations. The sensor formulation does not require any DNAzyme or hemin to function.

### Sensor Selectivity

First, we evaluated the selectivity of the sensor against metal cations other than Cu^2+^. For the selectivity assay, we chose to focus on transition metals that exist as divalent cations in aqueous solution and/or that are known to exhibit redox activity. Metals studied include Zn^2+^, Co^2+^, Ni^2+^, Mn^2+^, Fe^2+^, Fe^3+^, Pb^2+^, Hg^2+^, Cd^2+^, and Al^3+^. Chloride salts were used for all metals except Fe^2+^, which was added as ammonium iron sulfate (Mohr’s salt). We chose to use Mohr’s salt as the source of Fe^2+^ because FeCl_2_ was visually observed to begin precipitating almost immediately after dissolution in 18.2 MΩ water. An assay characterizing the sensor’s response to ammonium sulfate and FeCl_2_ can be found in Figure S6.

To perform the selectivity assay, lyophilized sensors were rehydrated with aqueous solution containing varying amounts of metal or with 18.2 MΩ water for control. The rehydrated sensors were plated, and the absorbance spectrum of each sensor was measured every 15 min for a total of five measurements including the zero-time measurement. It is important to note that approximately 5 min are required for absorbance spectra measurements, so that each interval between measurements is approximately 20 min.

The results of the selectivity assay, presented in Figure 1, show that the sensor is selective to Cu^2+^ against all other metal cations studied at concentrations up to 200 μM. In addition to absorbance measurements, photographs of sensor tubes were also taken at 15 min after rehydration to convey what an end-user of the sensor would observe visually were the sensor to be used in the field (Figure 1).

For assays involving the use of TMB oxidation for signal reporting, as is the case for this sensor, it is typical to monitor the absorbance peaks around 370 nm and/or 650 nm.^12^ However, there are two important considerations when reporting absorbance for our sensor. The first consideration is that the entire absorbance spectrum was observed to increase, especially at shorter wavelengths, when some metals were analyzed at high concentrations (ex. 2 mM Pb^2+^), even if there was no visually apparent blue color (Figure S7). The second is that our sensor exhibited an absorbance peak at approximately 680 nm (Figure 1a), indicating a shift to the typical absorbance peak around 650 nm. This shift is due to the presence of SDS in the sensor formulation (Figure S8). We identified that a strong correlation between visually observed blue color formation and absorbance could be achieved if the difference in absorbance between 680 and 500 nm for each spectral scan was used for quantification (Figures 1a and S8).

It should also be noted that a strong correlation between visually observed blue color formation and absorbance could also be achieved if the difference in absorbance between 390 and 500 nm for each spectral scan was used for quantification for studies using metal concentrations up to 200 μM. However, at metal concentrations of 2 mM, ΔAbs (390 – 500 nm) failed to produce as strong of a correlation as ΔAbs(680 – 500 nm) (Figure S8), which is why the metric ΔAbs(680 – 500 nm) was ultimately chosen.

### Correlating [Cu^2+^] with Absorbance Measurement

**Figure 2:**
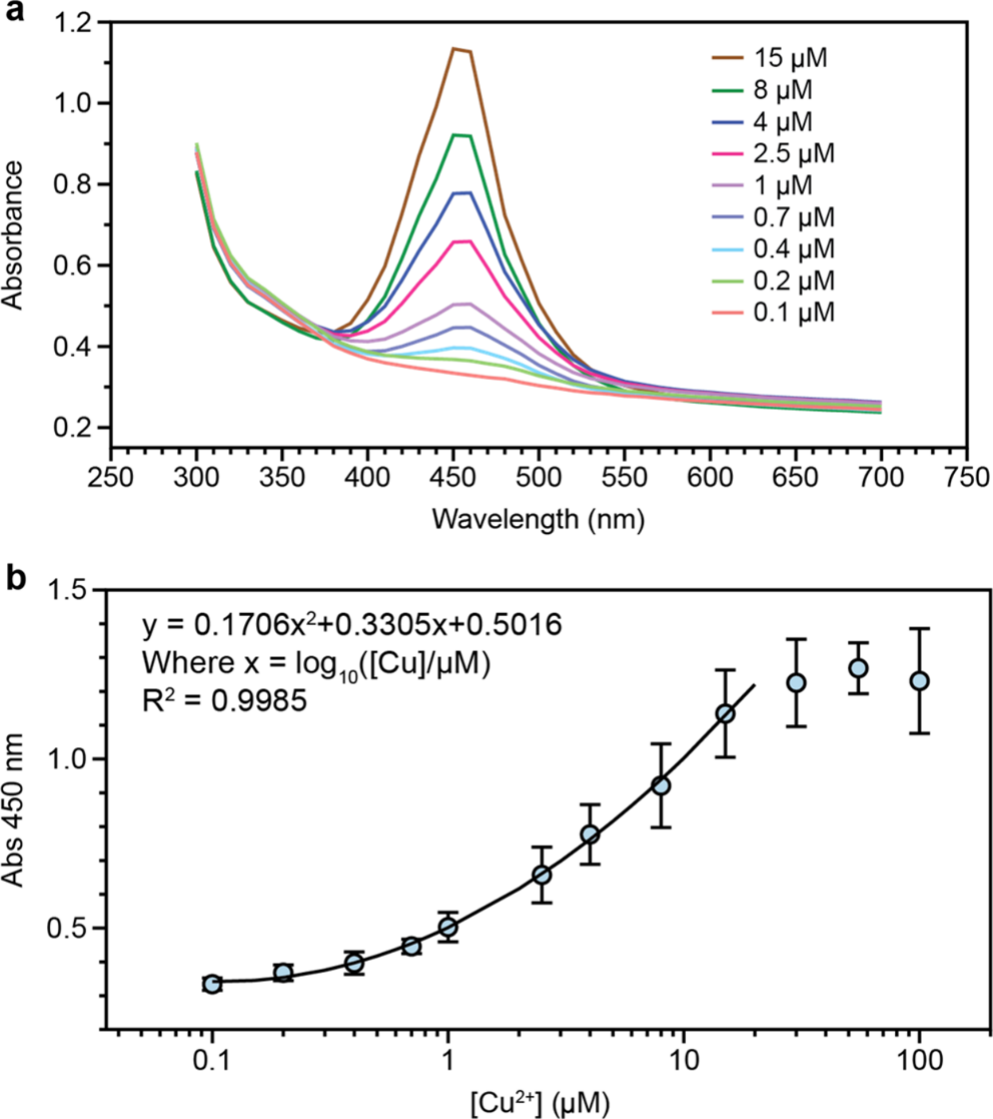
Correlating Cu^2+^ concentration with absorbance. (a) Absorbance spectra of the sensors after rehydrating them with different concentrations of Cu^2+^, incubating for 15 min at 25 °C, and then stopping the reactions with an equal volume of 2 M sulfuric acid. (b) Absorbance at 450 nm for the different concentrations of Cu^2+^ studied. A polynomial function was fit to the data on the range 0.1–15 μM as shown in the plot, where *x* = log_10_([Cu]) and [Cu] is in μM. Upon rehydration, sensor components are 10 mM NaH_2_PO_4_, 9.4 mM NaOH, 2.5 mM SDS, 0.75 mM TMB, 8 mM H_2_O_2_, and 2.2 M NaCl. Error bars are ± one standard deviation about the mean of three experimental replicates, each of which contained two technical replicates (*n* = 6).

Although we found that ΔAbs(680 – 500 nm) is generally correlated with Cu^2+^ concentration, we noted that ΔAbs(680 – 500 nm) changes over time (Figure 1), making it difficult to develop a quantitative correlation between Cu^2+^ concentration and absorbance. Therefore, to develop a quantitative correlation between Cu^2+^ concentration and absorbance, we rehydrated the lyophilized sensors as described previously but then after 15 min of incubation at 25 °C, we added an equal volume (20 μL) of 2 M sulfuric acid to stop the sensor reactions in accordance with previous studies.^11^ We then measured the absorbance spectra of the sensors and found that like previous studies,^11^ this approach allowed us to compute a nonlinear correlation (*R*^2^ = 0.9985) between absorbance at 450 nm and the log 10 of Cu^2+^ concentration over the range 0.1–15 μM (Figure 2).

### Sensor Threshold Tuning

For the sensor to be of practical use in the field, it should ideally produce visible color formation only when Cu^2+^ is at or above the US EPA limit of 20 μM (1.3 ppm) for copper in drinking water. Per the results displayed in Figure 1, it is obvious that the threshold concentration of Cu^2+^ at which the sensor activates is too low, with highly visible color formation occurring at 2 μM Cu^2+^. Therefore, we sought to tune the sensor’s activation threshold to achieve visible color formation only at or above 20 μM Cu^2+^. To accomplish this, we added varying amounts of diethylenetriamine-pentaacetic acid penta-sodium salt solution (DTPA) to understand its impact on the sensor’s activation threshold, as DTPA is known to chelate multivalent cations^13^ including Cu^2+^. The purchased DTPA stock, ∼40% purum in water, was calculated to be 1 M DTPA based on the manufacturer’s product data sheet. All stated DTPA concentrations were calculated on this basis.

**Figure 3:**
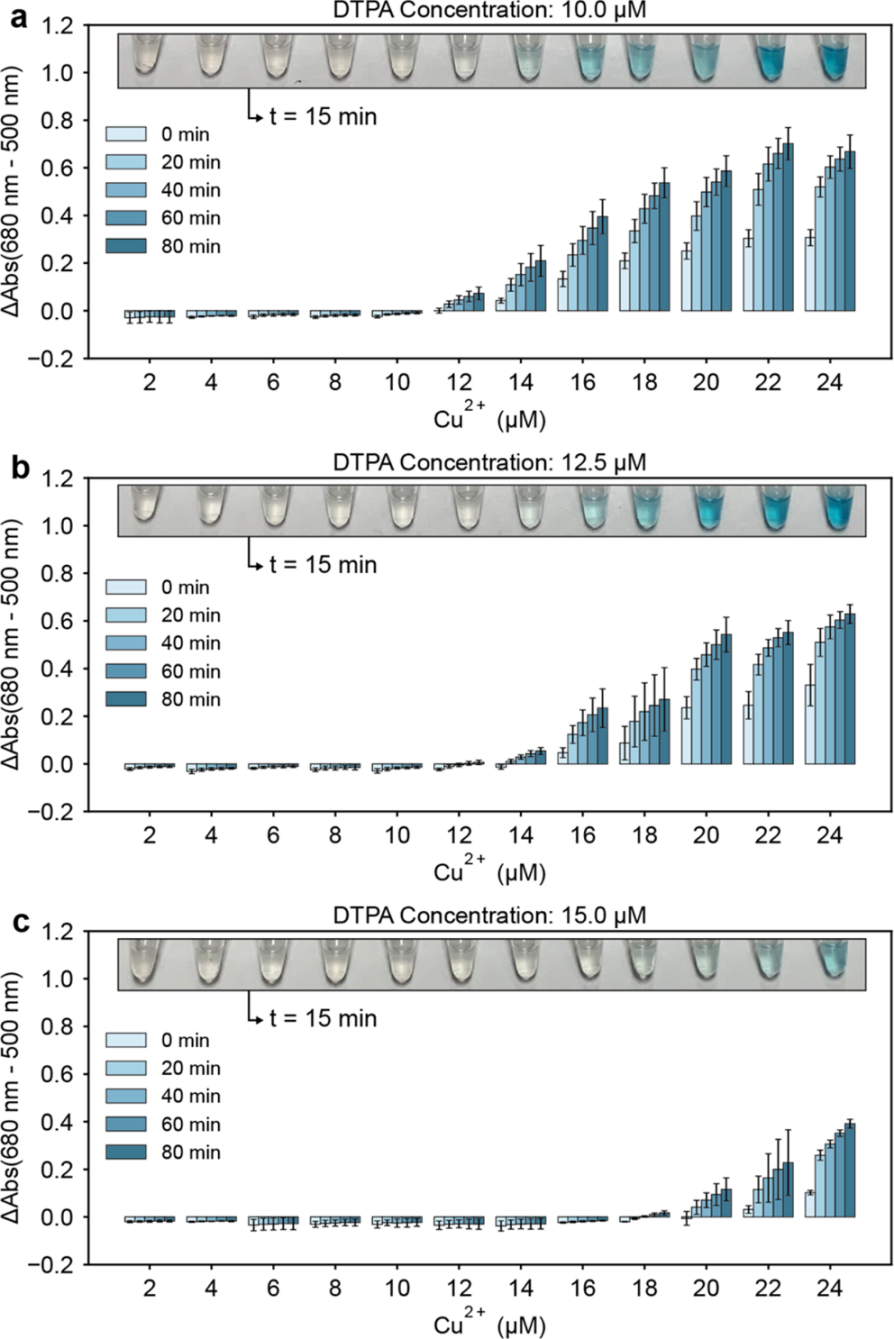
Sensor threshold tuning. Panels **a-c** present how the threshold concentration of Cu^2+^ at which the sensor produces blue color can be tuned via the addition of DTPA to the sensor formulation. Upon rehydration, sensor components are 10 mM NaH_2_PO_4_, 9.4 mM NaOH, 2.5 mM SDS, 0.75 mM TMB, 8 mM H_2_O_2_, 2.2 M NaCl, and the indicated DTPA concentration as stated in the plot titles. Photographs of sensor tubes are superimposed on each plot at 15 min after rehydration at 25 °C. Error bars are ± one standard deviation about the mean of three experimental replicates, each of which contained two technical replicates (*n* = 6).

The results, presented in Figure 3, show that the addition of DTPA to the sensor formulation increases the threshold concentration of Cu^2+^ at which visible color formation is observed. Based on the results of our threshold tuning experiments, we chose a DTPA concentration of 12.5 μM to achieve consistent, highly visible color formation near or above the US EPA limit of 20 μM copper, but little or no visible color formation at Cu^2+^ concentrations lower than the US EPA limit for copper.

### Sensor Function in Field Samples

**Figure 4:**
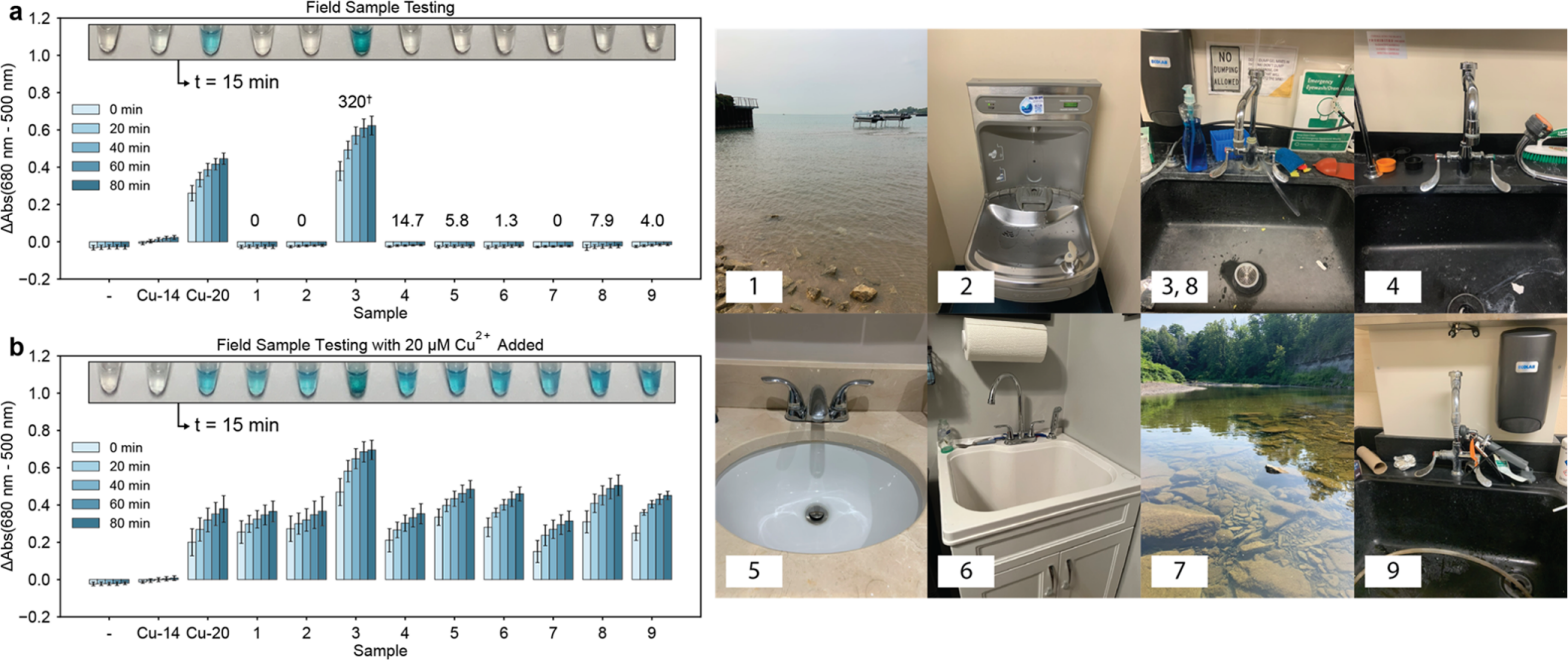
Sensor performance in field samples. (a) Field samples are labeled 1–9, with their origins depicted in the photographs. Copper concentrations of the field samples, measured via FAAS, are listed above the bar plots in μM. The copper concentration in field sample 3 was measured to be above the linear calibration range for FAAS (0–77 μM copper). (b) 20 μM Cu^2+^ was then added to each of the field samples and reanalyzed using the sensor. Upon rehydration, sensor components are 10 mM NaH_2_PO_4_, 9.4 mM NaOH, 2.5 mM SDS, 0.75 mM TMB, 8 mM H_2_O_2_, 2.2 M NaCl, and 12.5 μM DTPA. Photographs of sensor tubes are superimposed on each plot at 15 min after rehydration at 25 °C. Error bars are ± one standard deviation about the mean of three experimental replicates, each of which contained two technical replicates (*n* = 6). “-“ indicates rehydration with 18.2 MΩ water, while Cu-14 and Cu-20 indicate rehydration with 14 μM and 20 μM Cu^2+^ in 18.2 MΩ water, respectively. Field samples were sourced as follows with photographs taken by the authors: (1) Lake Michigan, Evanston, IL (2) water dispenser, Evanston, IL (3,8) sink sampled on two separate days, Evanston, IL (4) sink, Evanston, IL (5) sink, Evanston, IL (6) sink, Orchard Park, NY, (7) 18 Mile Creek, Derby, NY (9) sink, Evanston, IL.

Next, we gathered water samples from both municipal and environmental sources and tested them using the sensor formulation containing 12.5 μM DTPA, which is designed to produce a visible color near or above the US EPA limit of 20 μM copper. The results from field sample testing were compared to measurements of copper concentration using flame atomic absorption spectroscopy (FAAS). Full data and details around FAAS measurement can be found in Figure S9, Tables S1 and S2. The results, presented in Figure 4a, demonstrate the promising ability of the sensor to produce visible color only when copper concentration in the water is above 20 μM. To further evaluate the performance of the sensor, we then spiked 20 μM Cu^2+^ into each of the field samples and retested the samples using the sensor. The results, presented in Figure 4b, show that the sensor produced visible color for all field samples with 20 μM Cu^2+^ spiked into them.

### Sensor Shelf Life Study

To evaluate the shelf stability of the Cu^2+^ sensor, we performed a shelf life study where sensor performance was monitored for 12 days. Eleven sets of sensor tubes, one set for each day after day 0, were vacuum sealed with a desiccant card and stored in a dark cabinet at room temperature. The morning of each day of the study, the tubes were rehydrated with either 20 μM Cu^2+^ solution (made fresh on day 0 and day 7) or 18.2 MΩ water (control) and absorbance measured. One tube was kept aside and photographed 15 min after rehydration.

**Figure 5:**
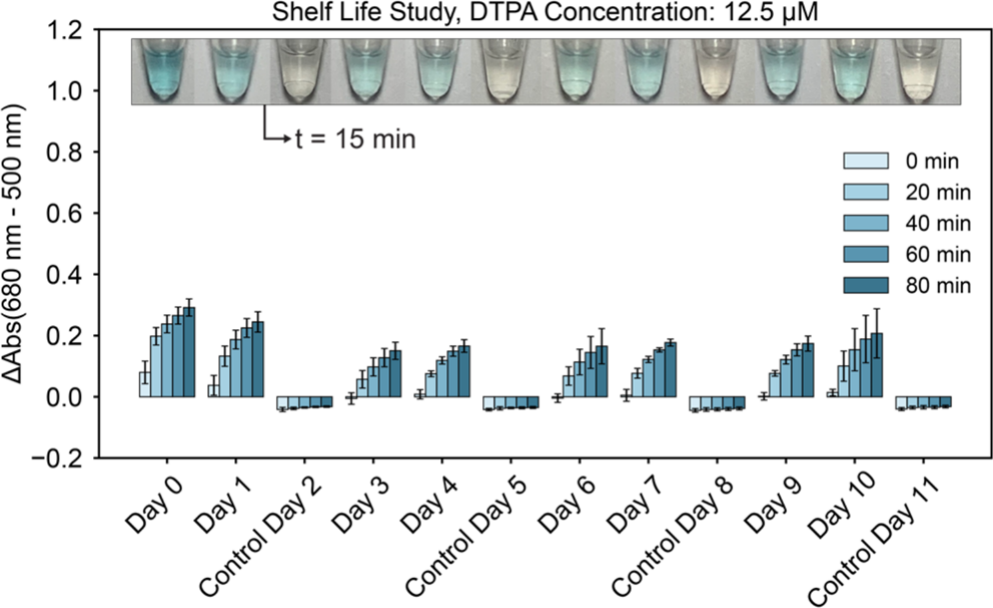
Sensor shelf stability. Upon rehydration, sensor components are 10 mM NaH_2_PO_4_, 9.4 mM NaOH, 2.5 mM SDS, 0.75 mM TMB, 8 mM H_2_O_2_, 2.2 M NaCl, and 12.5 μM DTPA. Photographs of sensor tubes are superimposed on each plot at 15 min after rehydration at 25 °C. Controls were rehydrated with 18.2 MΩ water on every third day; others were rehydrated with 20 μM CuCl_2_ solution, which was made fresh on day 0 and day 7. Error bars are ± one standard deviation about the mean of three experimental replicates, each of which contained two technical replicates (*n* = 6).

The results of the shelf life study, presented Figure 5, show that the sensor produced visible color formation when rehydrated with 20 μM Cu^2+^ throughout the entire study, and that the sensor did not produce visible color when rehydrated with 18.2 MΩ water. However, the speed at which color formation occurs and its intensity decrease from day 0 to day 3 and plateau thereafter. It is possible that shelf stability can be improved with further testing to identify the cause(s) of performance change between day 0 and day 3 and experimenting with formulation design such as including the use of lyoprotectants.

### Sensor Mechanism

**Figure 6:**
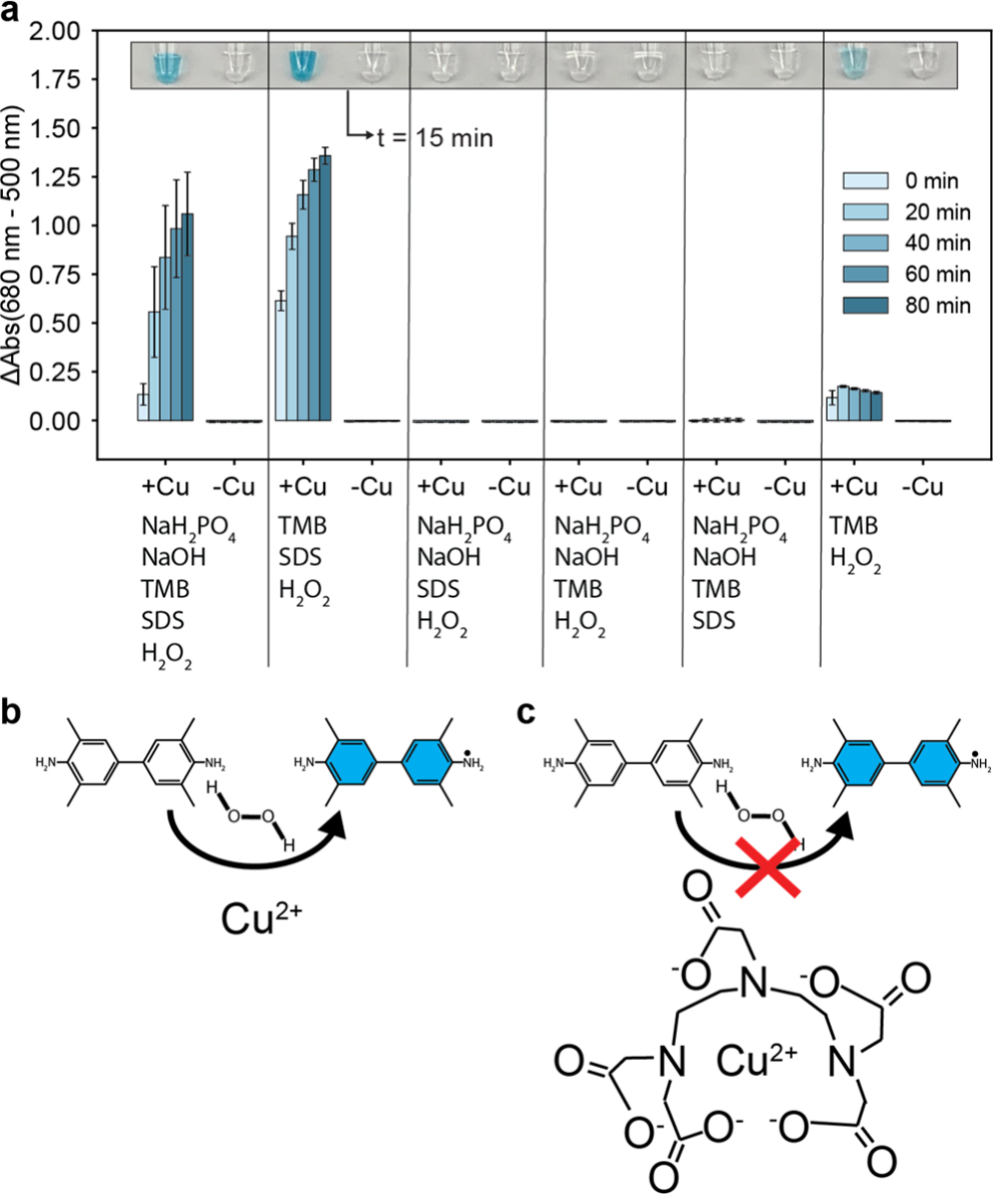
Mechanism of sensor function. (a) Nonlyophilized sensor reactions were assembled and absorbance measured at 25 °C to understand which components were required for blue color formation. The components present in each set of sensor reactions are listed in the plot. If present, the components were present at the following final concentrations: 10 mM NaH_2_PO_4_, 9.4 mM NaOH, 2.5 mM SDS, 0.75 mM TMB, and 8 mM H_2_O_2_. For each set of reactions, either 0 μM CuCl_2_ (–Cu) or 20 μM CuCl_2_ (+Cu) was present (final concentration). Photographs of sensor tubes are superimposed on each plot at 15 min after rehydration at 25 °C. Error bars are ± one standard deviation about the mean of three experimental replicates, each of which contained two technical replicates (*n* = 6). (b) Schematic representation of the Cu^2+^ driven oxidation of TMB. (c) Schematic representation of DTPA chelating Cu^2+^ to prevent its ability to drive TMB oxidation.

To investigate the sensor’s chemical mechanism, we performed an experiment to determine the minimum components required to achieve blue color formation. In this experiment, sensor reactions were assembled without lyophilization to avoid confounding the results of the study due to the lyophilization process itself. The results, presented in Figure 6a, show that only hydrogen peroxide, TMB, and Cu^2+^ are required to achieve blue color formation in a nonlyophilized sensor reaction. However, it is also apparent that the addition of SDS greatly enhances blue color formation.

Based on our findings and on prior literature, we hypothesize that Cu^*x*+^ participates in a Fenton-like reaction to generate reactive oxygen species from H_2_O_2_ and thereby drive the oxidation of TMB (Figure 6b), where *x* = 1, 2, or 3 depending on the stage in the catalytic cycle. Prior literature describes the ability of Cu^*x*+^ to participate in a Fenton-like reaction and oxidize organic substrates in the presence of H_2_O_2_,^14,15^ while TMB is known to become oxidized via the Fenton reaction.^15^ The cross-reactivity with iron II, the known catalyst in the Fenton reaction, further supports this hypothesis.

In addition, our finding that adding DTPA raises the threshold concentration of Cu^2+^ at which the sensor reaction takes place supports our hypothesis that Cu^*x*+^ participates in a Fenton-like reaction to drive the oxidation of TMB and yield blue color formation because DTPA is known to chelate Cu^2+^, and it is also known that Cu^2+^ chelators can be used to attenuate the Cu^*x*+^ driven Fenton-like reaction.^13,16^ Therefore, we hypothesize that the addition of DTPA to the reaction results in the chelation of Cu^2+^, thereby deactivating the ability of the Cu^2+^ that is chelated to participate in the Fenton-like reaction (Figure 6c).

Finally, our finding that SDS greatly increases the extent of blue color formation within our sensor reactions aligns with previous studies by Li et al., who demonstrated that the addition of SDS to horseradish peroxidase assays greatly enhances the extent of blue color formation when TMB is used as the chromogen.^17^ Li et al. hypothesize that SDS stabilizes the oxidized TMB molecule due to the electrostatic attraction between the positively charged TMB molecule and the negatively charged SDS.^17^ In addition, Li et al. hypothesize that SDS can enhance the solubility of TMB in solution, given that TMB is a nonpolar molecule.^17^ We believe that our findings corroborate the hypotheses of Li et al. and we speculate that SDS performs a similar function in our system including TMB solubilization and stabilization.

### Discussion

The sensor developed here demonstrates high selectivity to Cu^2+^ and visual limits of detection comparable to many colorimetric paper-based tests currently advertised for purchase.^18^ In addition, the sensor demonstrates functionality after 10 days of storage, with trends suggesting that longer shelf life is possible.

We believe the Cu^2+^ sensor has the potential for use in drinking water monitoring and monitoring of relatively clean environmental samples because the sensor demonstrated promising performance in the field samples studied here (Figure 4). In addition, we found that sensor functionality did not exhibit an appreciable dependence on temperature over a range of 25–40 °C (Figure S10).

Although the sensor has demonstrated promising performance, it has several potential limitations. First, the sensor may fail to detect copper species in the form of insoluble precipitates or in chelated form, as suggested by our study where DTPA was used to tune the threshold concentration of Cu^2+^ at which the sensor produced blue color (Figure 3). DTPA is known to chelate Cu^2+^, and we hypothesize that the chelation of Cu^2+^ by DTPA lowers the concentration of Cu^2+^ in solution that can drive TMB oxidation and thus color formation^13^ (Figure 6c).

In addition, the sensor’s performance may be impacted by samples with low or high pH (Figures S11 and S12), as both copper and phosphate speciation in solution are pH dependent. Similarly, DTPA protonation and its stability constant with Cu^2+^ are also pH dependent,^13,19^ thus potentially impacting sensor performance. Finally, it is common practice to add a strong acid to stop TMB oxidation in ELISA and other such assays.^20^ It is therefore possible that strongly acidic samples could inhibit TMB oxidation in the sensor formulation developed here.

Another consideration is the labile nature of the oxidized TMB species.^20^ This hurdle was overcome by the addition of SDS to the sensor formulation, which has been shown to to stabilize and/or encourage TMB color formation across a range of solution conditions.^21^ However, there may be a practical limit to which the oxidized chromophore species can be stabilized, such as at pH extremes.

Perhaps most importantly, the sensor formulation designed to activate at the US EPA limit of 20 μM copper may be prone to false positives when sufficiently high concentrations of interfering metals are present along with Cu^2+^ (Figures S13 and S14). Because DTPA is known to have a binding affinity for many different polyvalent cations^13,22^ other than Cu^2+^ and because the other cations studied do not activate the sensor in the absence of Cu^2+^ (Figures 1, S13, and S14), we hypothesize that such interfering metals could compete with Cu^2+^ for DTPA, thereby allowing an excess Cu^2+^ to remain unchelated, producing color formation at Cu^2+^ concentrations below the US EPA limit of 20 μM copper. Unfortunately, chelating agents with high binding specificity to Cu^2+^ are generally not commercially available.^23^ However, specialized chelating agents have been developed at lab scale to bind Cu^2+^ with much higher affinity than other metals.^23^ Use of such specialized chelating agents could possibly improve sensor performance by reducing the extent to which the chelator binds metals in solution other than Cu^2+^.

Finally, although we did not encounter any evidence that the disinfecting agents used in municipal water supplies interfere with sensor function, we recognize that the sensor may be prone to interference by disinfecting agents such as bleach at sufficiently high concentrations (Figure S15). We found that interference by bleach can be mitigated by pretreating the water sample with H_2_O_2_ and modifying the sensor format to include sodium bisulfite (Figure S15), though this pretreatment step would present a significant drawback from a user-friendliness standpoint in that an additional sample transfer step is required.

In summary, many additional tests are required to fully understand the capabilities and limitations of the sensor developed here. The best approach for understanding the utility of this sensor for monitoring drinking water quality would involve testing it against additional field samples and comparing results against standardized US EPA testing methods to better understand false positive/negative rates in a real-world setting.

## Conclusions

In this study, we developed a freeze-dried, field-deployable, colorimetric sensor for detecting aqueous Cu^2+^ in 15 min or less with a limit of detection of approximately 2 μM, well below the US EPA limit of 20 μM (1.3 ppm) for copper in drinking water. We demonstrate that the threshold concentration of Cu^2+^ at which the sensor activates can be tuned via the addition of a chelating agent so that color formation is only visually observed when Cu^2+^ concentration is near (within approximately 6 μM) or above 20 μM. We demonstrate sensor functionality in drinking water and environmental samples with various Cu^2+^ content and demonstrate satisfactory performance over a ten-day shelf life study.

Although promising, the sensor has some potential limitations. First, the sensor may be prone to false positives when sufficiently high concentrations of interfering metals are present along with Cu^2+^. Second, the sensor may fail to detect particulate copper or copper in chelated form. Third, sensor performance may be impacted by samples at either low or high pH extremes. Fourth, sensor performance may be impacted by solutions that contain oxidizing agents such as bleach.

Although a number of Cu^2+^ sensors have been described to date, we believe our sensor can provide some advantages. First, while many colorimetric sensors for Cu^2+^ employ specialized chemical probes produced via organic synthesis,^24−39^ our sensor is comprised entirely of commercially available reagents, simplifying sensor assembly. Second, we demonstrate how one can set a threshold response for the sensor to activate near and above the US EPA’s limit for copper to meet needs for drinking water testing, while many other sensors^40−43^ including some commercially available tests^18,44^ provide a colorimetric gradient across a wider range of Cu^2+^ concentrations, which may make it difficult to determine if copper concentration near (within 6 μM) or in excess of the US EPA’s limit of 1.3 ppm (20 μM). Third, we demonstrate that our sensor functions well across a temperature range of 25–40 °C. Fourth, our sensor demonstrates selectivity to Cu^2+^ against many other metal cations including Zn^2+^, Mg^2+^, Ca^2+^, Fe^3+^, Co^2+^, Ni^2+^, Mn^2+^, Al^3+^, Pb^2+^, Hg^2+^, Cd^2+^ at concentrations up to 2000 μM, and selectivity against Fe^2+^ at concentrations up to 200 μM. Fifth, we demonstrate that our sensor can be assembled and stored for several days before being deployed for use in a just-add-water format. Finally, our sensor provides colorimetric results visible to the naked eye in 15 min or less. In summary, we believe the sensor formulation presented here can provide some advantages compared to other technologies in its simplicity and user-friendliness.

## Supporting Information Available

Detailed instructions on sensor assembly; information on the absorbance metric used; additional sensor analysis including interfering effects; FAAS data (PDF)
Sensor assembly flowsheets; raw data (ZIP)

## Abbreviations

US: United States
EPA: Environmental Protection Agency
LCR: Lead and Copper Rule
PoN: point-of-need
DTPA: diethylenetriamine-pentaacetic acid penta-sodium salt
ACS: American Chemical Society
SKU: stock keeping unit
TMB: 3,3′,5,5′-Tetramethylbenzidine
SDS: sodium dodecyl sulfate
PCR: polymerase chain reaction
FAAS: flame atomic absorption spectroscopy
