# Supplementary material for: A Sensor for Detecting Aqueous Cu2+ That Functions in a Just-Add-Water Format
Source: ACS Omega. 2024 Dec 19;10(1):1188–97. doi: 10.1021/acsomega.4c08751 (PMC11740102; doi:10.1021/acsomega.4c08751)
Supplement: Supplementary file 1 — ao4c08751_si_001.pdf [file ao4c08751_si_001.pdf]

Supporting Information for: A Sensor for Detecting Aqueous Cu^{2+} that Functions in a Just-Add-Water Format

Tyler J. Lucci[a,b,c], Abigail Neufarth[d†], Jean-François Gaillard[d], and Julius B. Lucks[a,b,c,e,f]*

[a]Department of Chemical and Biological Engineering, Northwestern University, 2145 Sheridan Road, Evanston, IL 60208, United States

[b]Center for Synthetic Biology, Northwestern University, 633 Clark Street, Evanston, IL 60208, United States

[c]Center for Water Research, Northwestern University, 2205 Tech Drive, Evanston, IL 60208, United States

[d]Department of Civil and Environmental Engineering, 2145 Sheridan Road, Northwestern University, Evanston, IL 60208, United States

[e]Interdisciplinary Biological Sciences Graduate Program, 2205 Tech Drive, Northwestern University, Evanston, IL 60208, United States

[f]Chemistry of Life Processes Institute, Northwestern University, 2170 Campus Drive, Evanston, IL 60208, United States

Sensor Assembly Procedure

The following provides detailed instructions for assembling a batch of 24 sensors. **Supplementary Flowsheets** provide additional information on reagent dilutions, solution assembly, and solution additions.

First, dry ice is obtained, and an aluminum tube holder is placed on the dry ice for pre-cooling (**Figure S1**). Next, a calendar pin is used to puncture three holes in the caps of each PCR tube (Bio-Rad 0.2 mL Cat. No. TFI0201) to be used (**Figure S1**). The PCR tubes are then placed inside a plastic tube holder at room temperature (**Figure S1**). 30% hydrogen peroxide is then serially diluted with 18.2 MΩ water to 10X the desired final concentration (80 mM) in 1.5 mL snap-top tubes (Posi-Click Mfr. No. C2170). 2 µL of the 10X hydrogen peroxide solution is then added via pipette to each PCR tube.

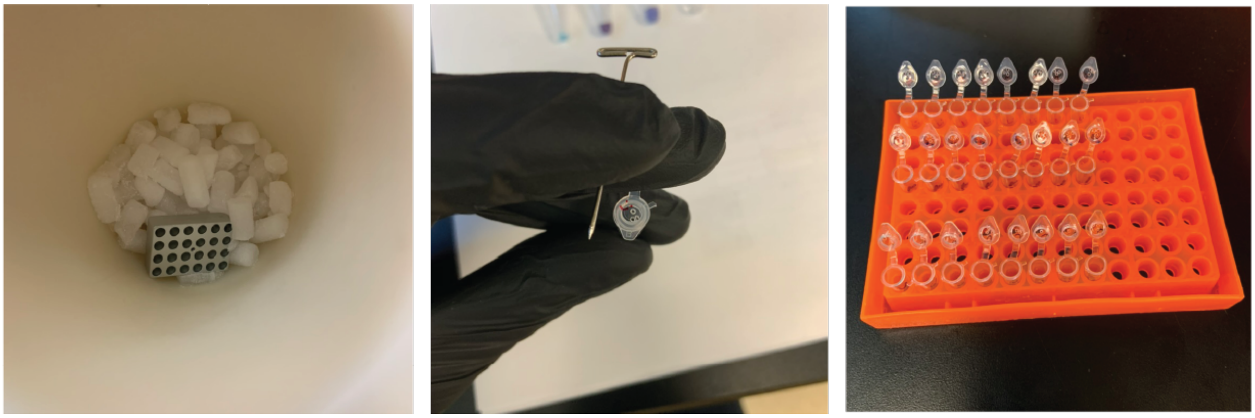

Figure S1: Preparing materials for sensor assembly. **Left**: an aluminum block is pre-cooled on dry ice. **Middle**: PCR tube caps are punctured using a calendar pin. **Right**: PCR tubes are placed inside a plastic tube holder at room temperature.

Next, 200 µL (or 192 if adding DTPA) of buffer solution is made. To make buffer solution, 149.5 µL of 18.2 MΩ water, 8 µL of 1 M sodium phosphate monobasic, 20 µL of 0.1 M sodium dodecyl sulfate, and 7.5 µL of 1 M sodium hydroxide are added to a 1.5 mL snap-top tube (Posi-Click Mfr. No. C2170), in that order, and then vortex mixed for a few seconds. 15 µL of 40 mM TMB (in DMSO) is then added to the buffer mixture, and the solution is again vortex mixed for a few seconds.

To make the 480 µL of reaction premix (at room temperature) 330 µL of 4 M sodium chloride is added to a 1.5 mL snap-top tube, followed by an addition of 150 µL of buffer solution. The reaction premix is then vortex mixed for twenty to thirty seconds (**Figure S2**). Then, 16 µL of the reaction premix is added to each PCR tube with pipette mixing to ensure that the 16 µL of reaction premix is thoroughly mixed with the existing 2 µL of oxidant solution previously added to the tubes. After adding the reaction premix, the PCR tube is placed in the pre-cooled aluminum tube holder that is still on dry ice (**Figure S2**).

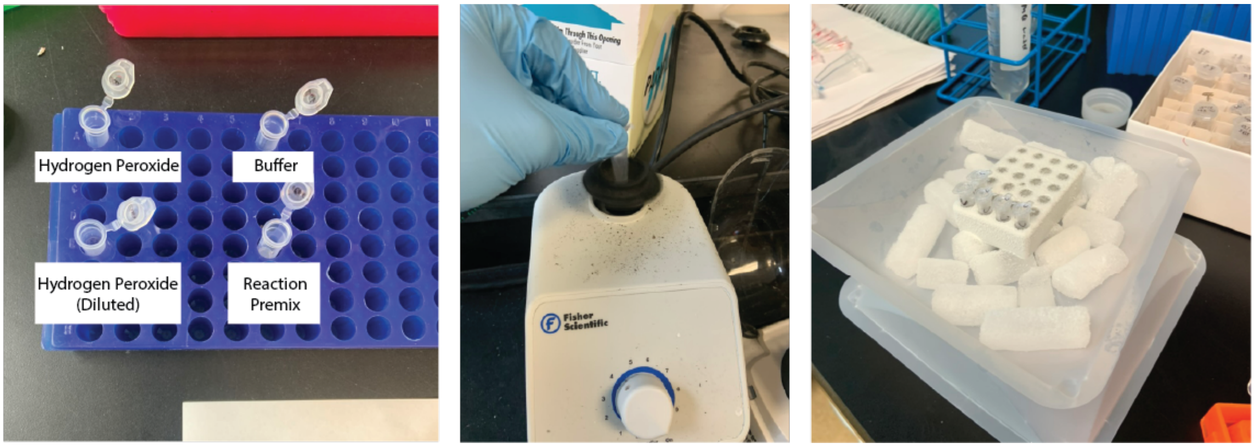

Figure S2: Assembling sensors. **Left**: an example layout of snap-top tubes for preparing the solutions required for sensor assembly. **Middle**: vortex mixing solutions to ensure homogeneity. **Right**: once the sensor is assembled, the PCR tube is immediately placed in the aluminum tube holder that has been pre-cooled on dry ice.

Once 24 PCR tubes are ready, the aluminum tube holder is wrapped in aluminum foil and a small tear is made in the bottom to allow liquid nitrogen drainage (**Figure S3**). The foil-wrapped block is then submerged inside liquid nitrogen for five minutes or until vigorous bubbling stops (**Figure S3**). After cooling the block to liquid nitrogen temperature, the block is withdrawn from the liquid nitrogen using tweezers and the liquid nitrogen is drained through the hole in the foil (**Figure S3**). The aluminum tube holder is then partially unwrapped to expose the tops of the tubes, and then placed inside a glass beaker for lyophilization (**Figure S3**).

For lyophilization, a FreeZone 2.5 L -84 °C lyophilizer by Labconco is used. Prior to lyophilizing the sensors, the collector is turned on and allowed to cool to approximately -84 °C. Then, the vacuum pump is turned on at a setpoint of 0.04 mbar. Finally, the glass beaker with aluminum tube holder is attached to the lyophilizer stem and valve opened slowly to pull vacuum.

The sensors are lyophilized for 16 hours. Properly lyophilized sensors should form a pellet at the bottom of the PCR tube (**Figure S3**). If lyophilizing multiple batches of tubes at once, it is best to cool all tubes in liquid nitrogen and load them onto the lyophilizer all at once rather than place additional batches onto the lyophilizer when other tubes are partially completed. Placing batches of tubes on the lyophilizer while others are running results in pressure swings that may affect the lyophilization process.

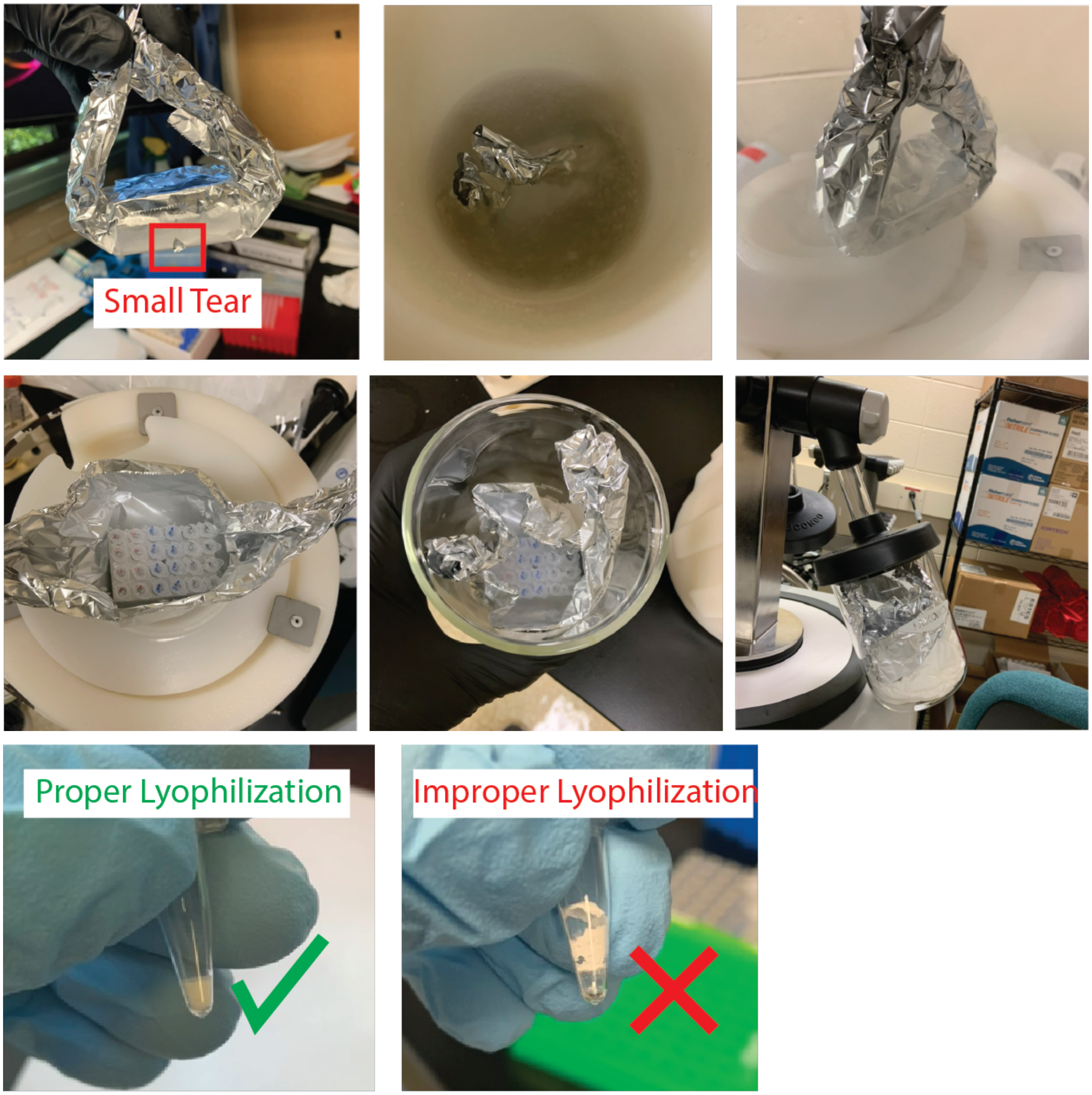

Figure S3: Lyophilizing the sensors. The pre-cooled sensors inside the aluminum block are wrapped in foil, cooled in liquid nitrogen, partially unwrapped, and then lyophilized. Properly lyophilized sensors will form a pellet at the bottom of the tube. Improperly lyophilized sensors will form a disorganized, puffy structure.

When additives are used, such as the chelating agent diethylenetriamine-pentaacetic acid penta-sodium salt (DTPA), the amount of water added to formulate the buffer solution is reduced from 149.5 µL to 141.5 µL, bringing the total volume of buffer solution to 192 µL. To formulate 480 µL of reaction premix in this case, 330 µL of 4 M sodium chloride is added to a 1.5 mL snap-top tube, followed by an addition of 6 µL of the desired additive (at 100X the desired final concentration), followed by 144 µL of buffer solution. The reaction premix is then vortex mixed for twenty to thirty seconds and then dosed into PCR tubes.

Supplementary Figures

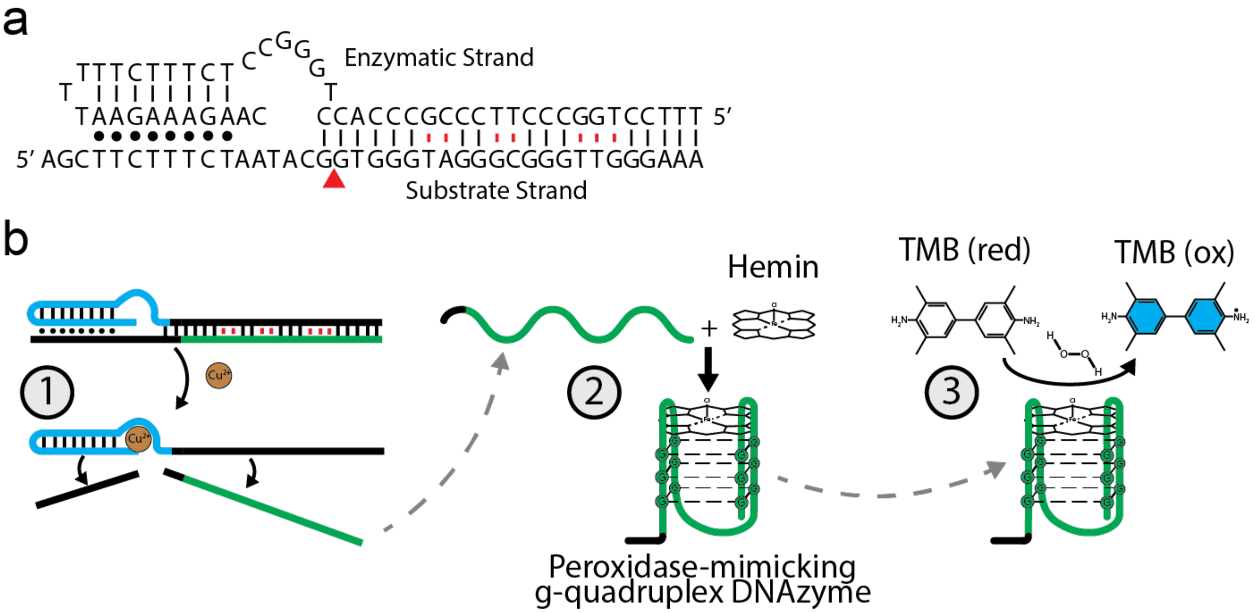

Figure S4: Initial strategy for developing a colorimetric sensor for detecting Cu^{2+} in drinking water that functions in a single tube format. (**a**) Cu^{2+} dependent DNAzyme design that includes base pair mismatches (red lines) to lower the melting temperature of enzyme-substrate complex post-cleavage. The cleavage point is marked with a red triangle. (**b**) In this system, the Cu^{2+} dependent DNAzyme would cleave its substrate strand to yield a single-stranded DNA oligo with repeating g-triplicates (1) that could then complex with hemin to form the peroxidase-mimicking g-quadruplex DNAzyme (2) and drive the oxidation of TMB in the presence of hydrogen peroxide to yield a colorimetric output (3).

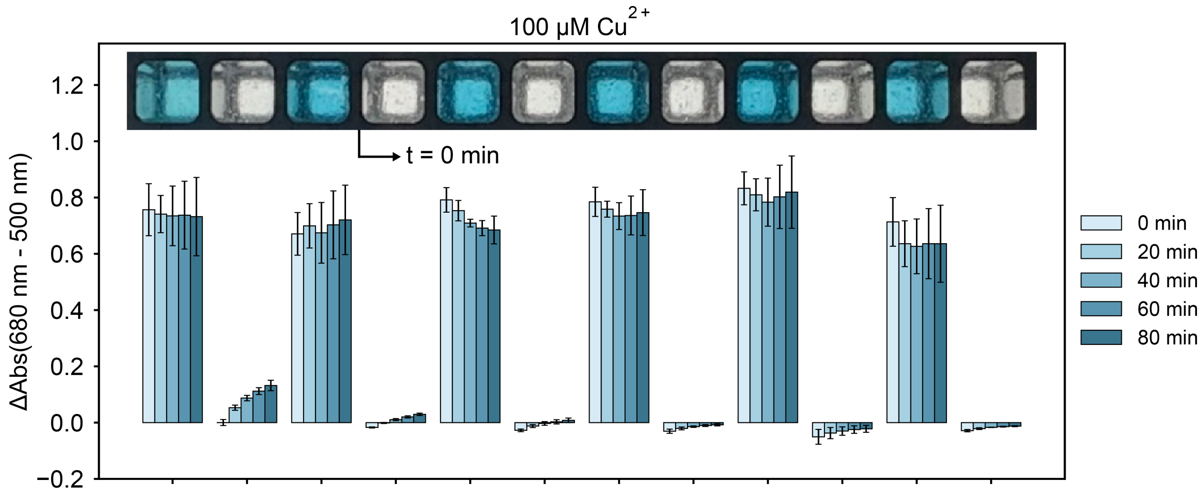

Cu^{2+} (μM)	100	0	100	0	100	0	100	0	100	0	100	0
DNAzyme (nM)	500		0		500		0		500		0	
Hemin (μM)	1				0.1				0			

Figure S5: The presence of Cu^{2+} alone is sufficient to result in blue color formation without any requirement for hemin or the peroxidase-mimicking g-quadruplex DNAzyme at 25 °C. Sensor reactions contained final concentrations of 1X phosphate buffered saline (PBS) (137 mM NaCl, 2.7 mM KCl and 11.9 mM phosphates, pH 7.4), 2.5 mM SDS, 0.5 mM TMB, 1 mM hydrogen peroxide, 1500 mM NaCl (in addition to that from the PBS), variable hemin (1 µM, 0.1 µM, 0 µM as indicated), variable DNAzyme substrate strand (500 nM or 0 nM as indicated, annealed to the enzymatic strand at a ratio of 1:1.1 substrate:enzymatic strand), and variable $CuCl_2$ (100 µM or 0 µM as indicated). Prior to assembling the sensor, the enzymatic and substrate DNAzyme strands (PAGE gel purified) were annealed in HEPES buffer pH 7 with 1500 mM NaCl by heating to 95 °C and cooling to 25 °C and then further cooling on ice at a ratio of 1:1.1 substrate to enzymatic strand. **Supplementary Flowsheets** provide additional information on reagent dilutions, solution assembly, and solution additions. A photograph of a representative plate immediately before absorbance measurement is presented to show visually apparent blueness. Error bars are ± one standard deviation about the mean of three experimental replicates, each of which contained two technical replicates (n = 6).

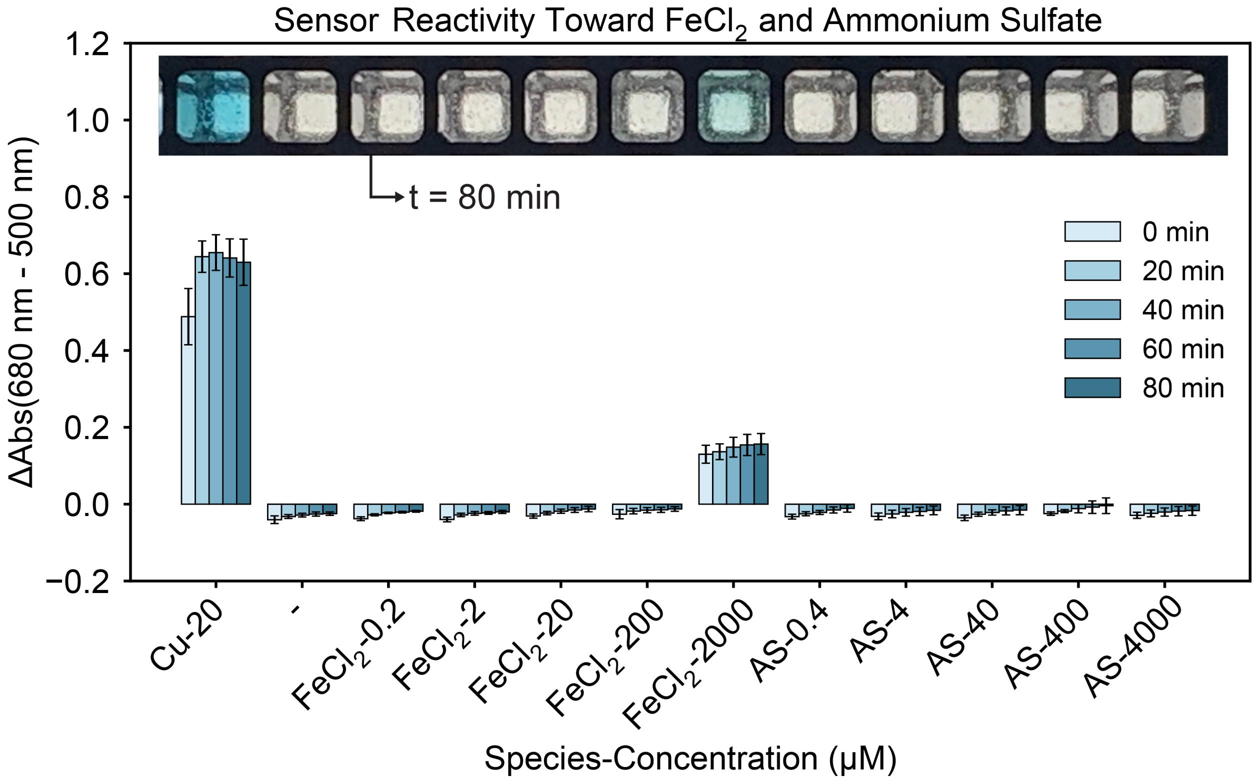

Figure S6: Reactivity of the sensor toward iron (II) chloride (“$FeCl_2$”) and ammonium sulfate (“AS”) at 25 °C. Labeling scheme denotes species–concentration, where concentration is in µM. For example, “$FeCl_2$-0.2” indicates rehydration with 0.2 µM $FeCl_2$. “–“ indicates rehydration with 18.2 MΩ water. Upon rehydration, sensor components are 10 mM NaH_2PO_4, 9.4 mM NaOH, 2.5 mM SDS, 0.75 mM TMB, 8 mM H_2O_2, and 2.2 M NaCl. A photograph of a representative plate immediately after absorbance measurement is presented to show visually apparent blueness. Error bars are ± one standard deviation about the mean of three experimental replicates, each of which contained two technical replicates (n = 6).

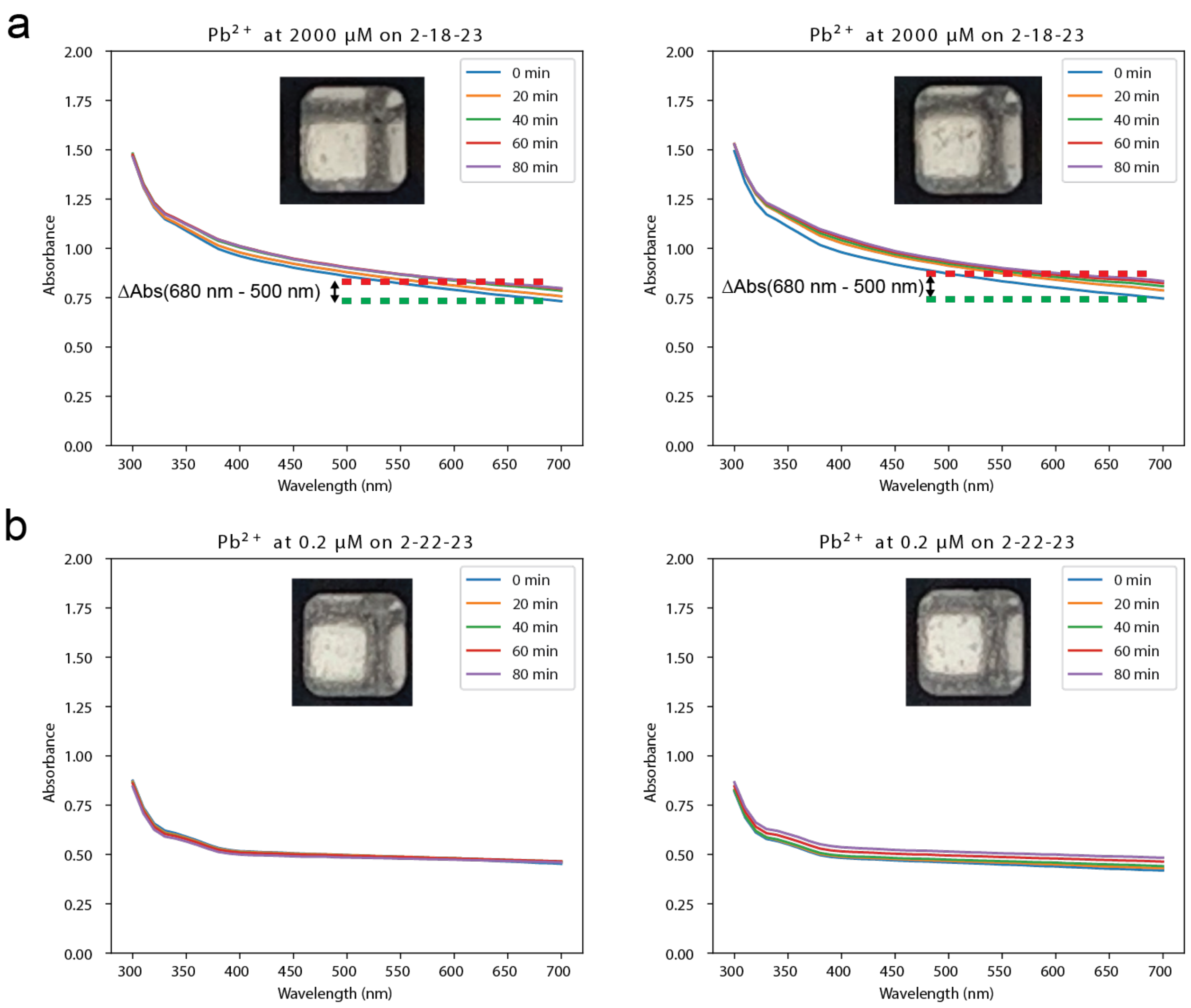

Figure S7: Changing of the absorbance spectra as Pb^{2+} concentration increases at 25 °C. Panels **a-b** show how the absorbance spectra, including absorbance at 680 nm, of sensors rehydrated with Pb^{2+} increase at high (2 mM) Pb^{2+} concentration without visually apparent blueness. Each plot corresponds to a single technical replicate. Photographs taken immediately before absorbance measurement of the four cells for which absorbance data are reported are provided as insets in each plot. Upon rehydration, sensor components are 10 mM NaH_2PO_4, 9.4 mM NaOH, 2.5 mM SDS, 0.75 mM TMB, 8 mM H_2O_2, and 2.2 M NaCl. In some cases, the absorbance spectrum can change such that the absorbance metric used throughout, ΔAbs(680 nm – 500 nm), becomes negative, as emphasized in panel **a**.

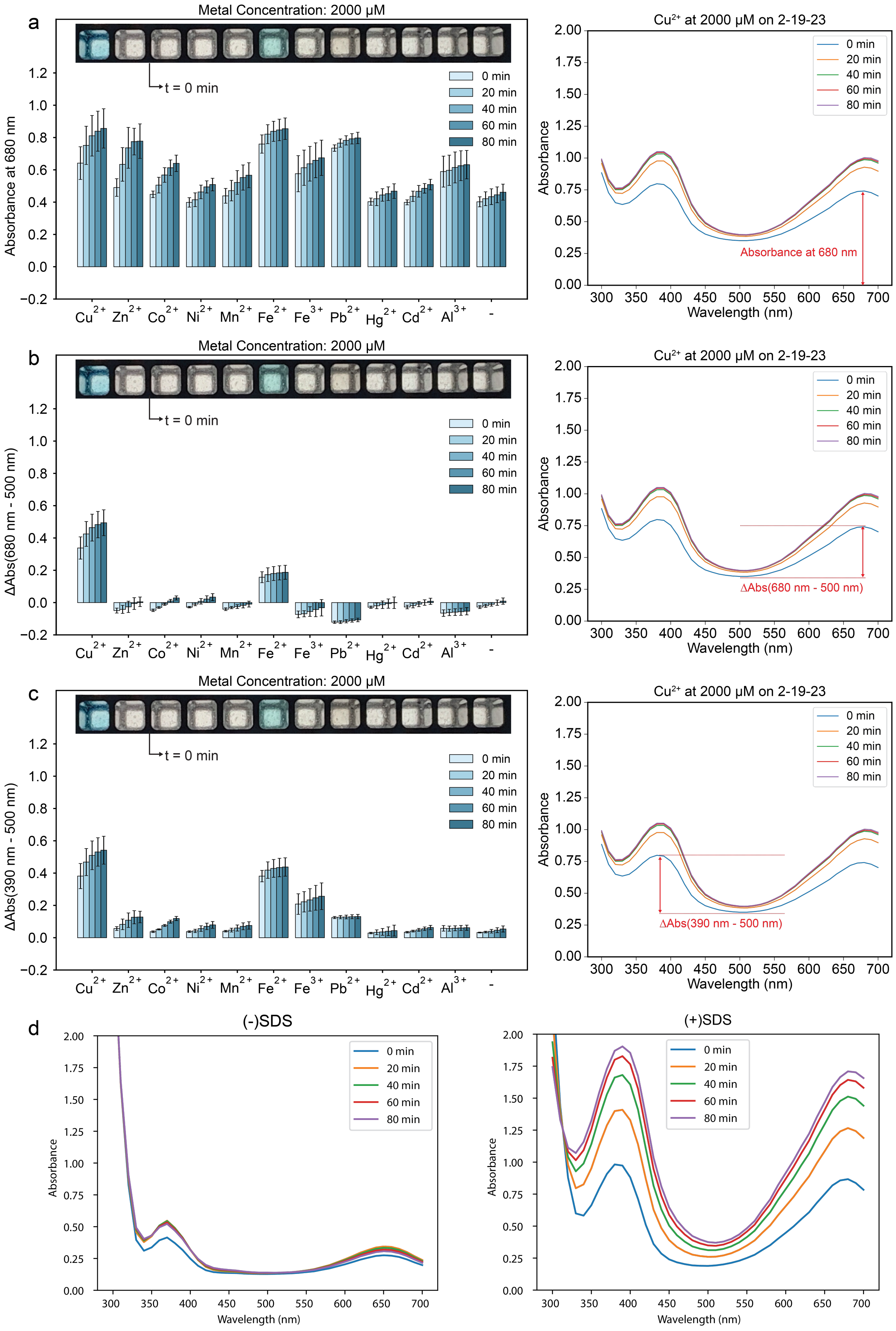
a
b
c
d
Metal Concentration: 2000 μM
t = 0 min
0 min
20 min
40 min
60 min
80 min
Absorbance at 680 nm
ΔAbs(680 nm - 500 nm)
ΔAbs(390 nm - 500 nm)
Cu2+ Zn2+ Co2+ Ni2+ Mn2+ Fe2+ Fe3+ Pb2+ Hg2+ Cd2+ Al3+ -
Cu2+ at 2000 μM on 2-19-23
Absorbance
Wavelength (nm)
Absorbance at 680 nm
ΔAbs(680 nm - 500 nm)
ΔAbs(390 nm - 500 nm)
(-)SDS
(+)SDS

Figure S8: Methods of quantifying blue color formation for the sensor. Panels **a**, **b**, and **c** present different methods of quantifying blue color formation via absorbance measurement for the same set of data, while panel **d** presents differences in absorbance peaks in the presence and absence of SDS. Panel **a** presents raw absorbance at 680 nm, while panel **b** presents absorbance at 680 nm minus absorbance at 500 nm, which is the metric used in the manuscript. Panel **c** presents absorbance at 390 nm minus absorbance at 500 nm. A photograph of a representative plate immediately before absorbance measurement is presented in both panels to convey visually apparent blueness. Panel **a** does not correlate well with visually apparent blueness, while panel **b** does correlate well with visually apparent blueness. Panel **c** correlates reasonably well with visually apparent blueness; however, the Fe^{3+} condition shows an increase in ΔAbs(390 nm – 500 nm) without visually apparent blueness. Therefore, ΔAbs(680 nm – 500 nm) was chosen as the absorbance metric in the manuscript. In some cases, such as for 2000 µM Pb^{2+}, the absorbance metric ΔAbs(680 nm – 500 nm) can become negative. This is due to changes in the absorbance spectrum as presented in **Figure S7**, which we hypothesize could be due to the formation of metal precipitates and associated light scattering. Panel **d** shows that when SDS is added to the sensor formulation, the absorbance peak at 650 nm shifts to 680 nm (left: 0.75 mM TMB, 8 mM H_2O_2, 20 µM Cu^{2+}, right: 0.75 mM TMB, 2.5 mM SDS, 8 mM H_2O_2, 20 µM Cu^{2+} (data sourced from the mechanism study experiment presented as **Figure 6** in the manuscript). In panels **a-c**, error bars are ± one standard deviation about the mean of three experimental replicates, each of which contained two technical replicates (n = 6).

Cu^{2+} FAAS Measurement and Results

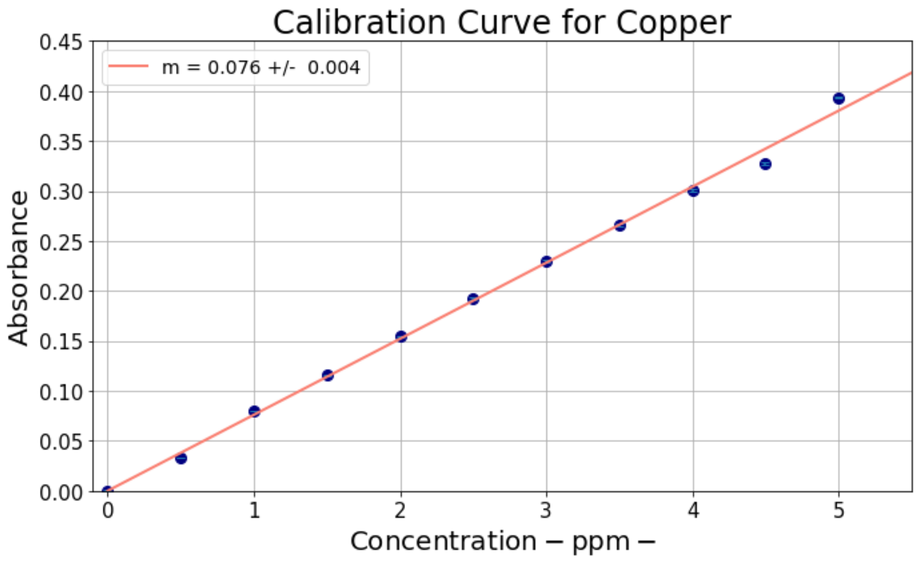

Figure S9: Calibration plot for copper measurement via FAAS. Error bars represent one standard deviation about the mean of each point but are not visible because of their small magnitudes. The calibration slope (m) is provided in the plot legend. Slope uncertainty was computed using the value of a Student's t test for a continuous random variable (t), the residual sum of squares using the best fit slope (RSS), degrees of freedom (m), and sample corrected sum of squares (Sxx) as outlined below in Equation 1.

1. $\text{slope uncertainty} = t \cdot \frac{\sqrt{\frac{RSS}{m}}}{\sqrt{S_{XX}}}$ where $RSS = \sum(y_i - mx_i)^2$ and $m = n - 2, S_{XX} = \sum(x_i - \bar{x})^2$

Sample	Cu Concentration (ppm)	Cu Concentration (μM)	pH
Sample 1	BQL	BQL	8.6
Sample 2	BQL	BQL	8.6
Sample 4	0.94+/-0.05	14.7+/-0.8	8.3
Sample 5	0.368+/-0.019	5.80+/-0.30	8.5
Sample 6	0.082+/-0.004	1.29+/-0.07	8.5
Sample 7	BQL	BQL	8.4
Sample 8	0.504+/-0.026	7.9+/-0.4	8.4
Sample 9	0.251+/-0.013	3.95+/-0.21	8.6
Sample 3	20.2+/-1.1	319+/-17	8.5

Table S1: FAAS and pH measurements for the field samples tested with the Cu^{2+} sensor, the results of which are presented in **Figure 4a**. For some samples, the Cu concentration is listed as BQL for "below quantitation limit" to denote that the Cu concentration of the sample was measured to be below the lower limit of quantitation of approximately 20 ppb (315 nM).

Sample	Cu Concentration (ppm)	Cu Concentration (μM)	pH
Sample A	0.101+/-0.006	1.60+/-0.09	1.8
Sample B	0.79+/-0.04	12.4+/-0.6	1.7
Sample C	1.85+/-0.10	29.2+/-1.5	1.7
Sample E	2.98+/-0.15	46.8+/-2.4	1.2
Sample F	0.342+/-0.018	5.38+/-0.28	0.9
Sample G	BQL	BQL	1.0
Sample H	2.19+/-0.11	34.5+/-1.8	0.9
Sample I	BQL	BQL	0.9
Sample J	1.06+/-0.06	16.7+/-0.9	0.9

Table S2: FAAS and pH measurements for the acidified field samples tested with the Cu^{2+} sensor, the results of which are presented in **Figure S12**. For some samples, the Cu concentration is listed as BQL for "below quantitation limit" to denote that the Cu concentration of the sample was measured to be below the lower limit of quantitation of approximately 20 ppb (315 nM).

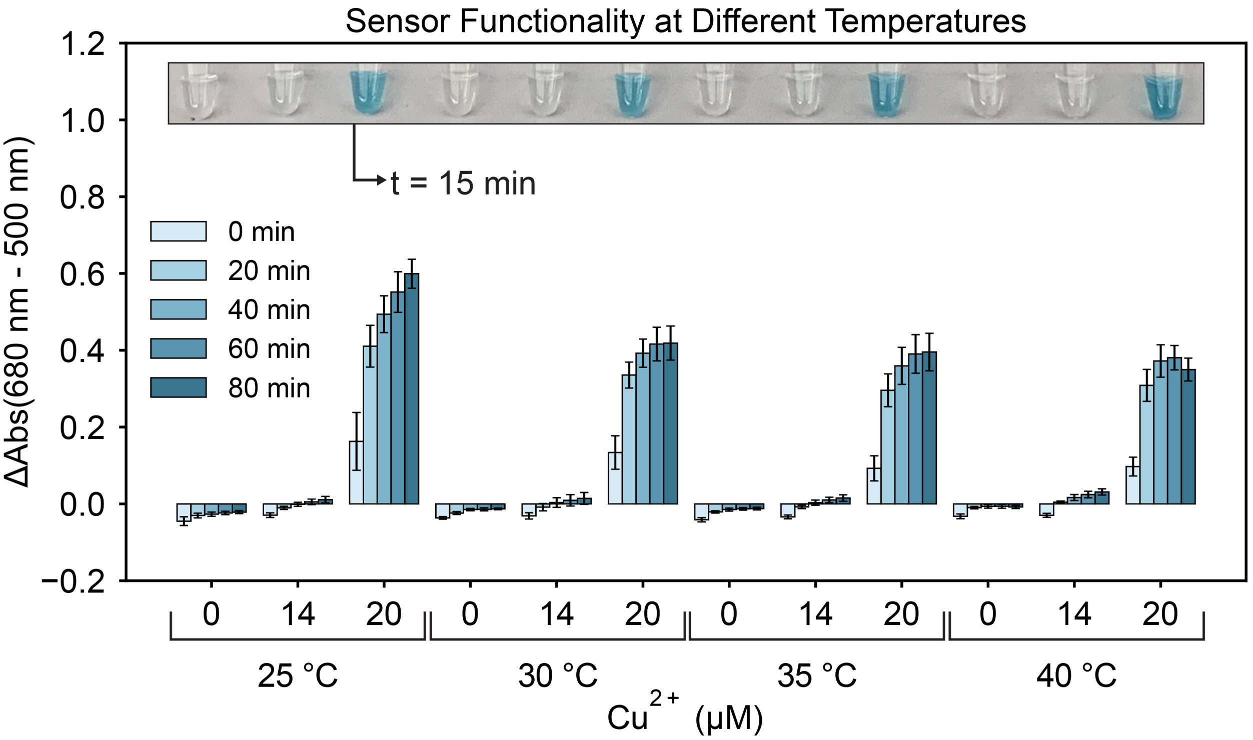

Figure S10: Impact of temperature on sensor functionality. To study the impact of temperature on sensor functionality, sensors were rehydrated with 18.2 MΩ water containing either 0 µM, 14 µM, or 20 µM $CuCl_2$. These sensors were then plated, and absorbance measured as described in the Absorbance Measurement section with the exception that absorbance measurements and associated incubations were performed at the temperatures listed in the plot (25 °C, 30 °C, 35 °C, and 40 °C). Upon rehydration, sensor components are 10 mM NaH_2PO_4, 9.4 mM NaOH, 2.5 mM SDS, 0.75 mM TMB, 8 mM H_2O_2, 2.2 M NaCl, and 12.5 µM DTPA. Photographs of sensor tubes are superimposed on each plot at 15 minutes after rehydration. Error bars are ± one standard deviation about the mean of three experimental replicates, each of which contained two technical replicates (n = 6).

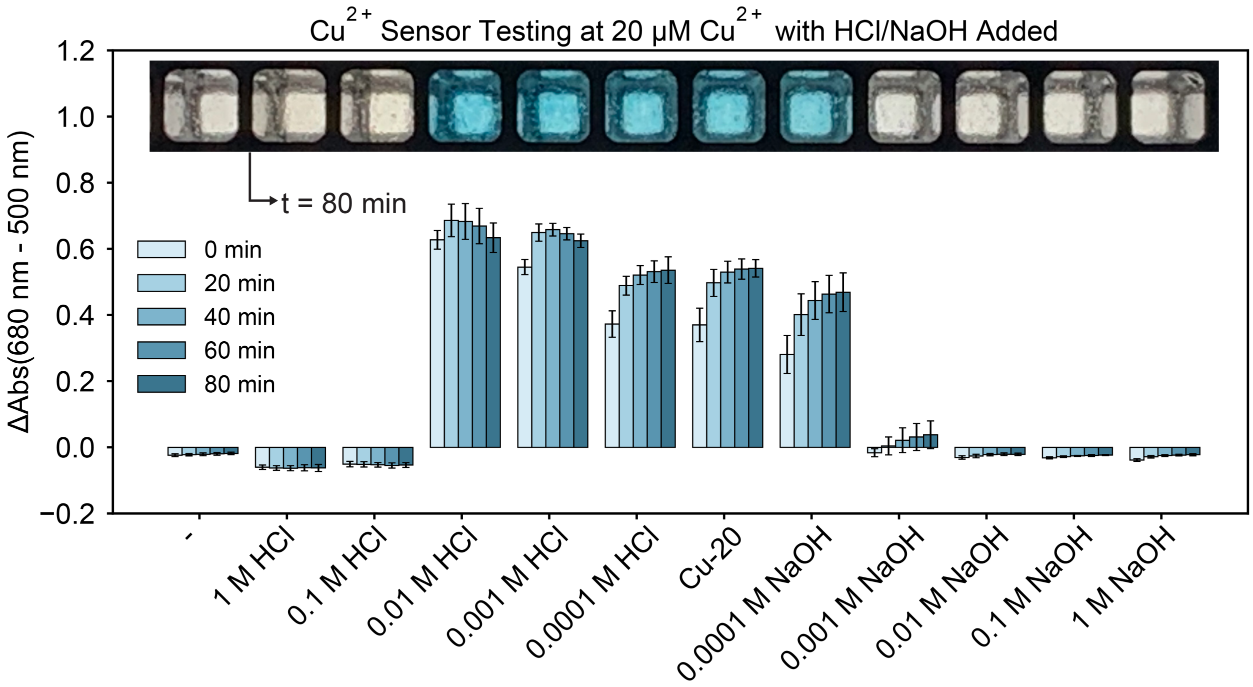

Figure S11: Sensor performance in acidic (HCl) and basic (NaOH) samples at 25 °C. All samples, except for the 18.2 MΩ water blank, contain 20 µM Cu^{2+} and the concentration of acid or base stated in the plot. "–" indicates rehydration with 18.2 MΩ water, while "Cu-20" contains 20 µM Cu^{2+} in 18.2 MΩ water with no added acid or base. Upon rehydration, sensor components are 10 mM NaH_2PO_4, 9.4 mM NaOH, 2.5 mM SDS, 0.75 mM TMB, 8 mM H_2O_2, and 2.2 M NaCl. A photograph of a representative plate immediately after absorbance measurement was completed is presented to show visually apparent blueness. Error bars are ± one standard deviation about the mean of three experimental replicates, each of which contained two technical replicates (n = 6).

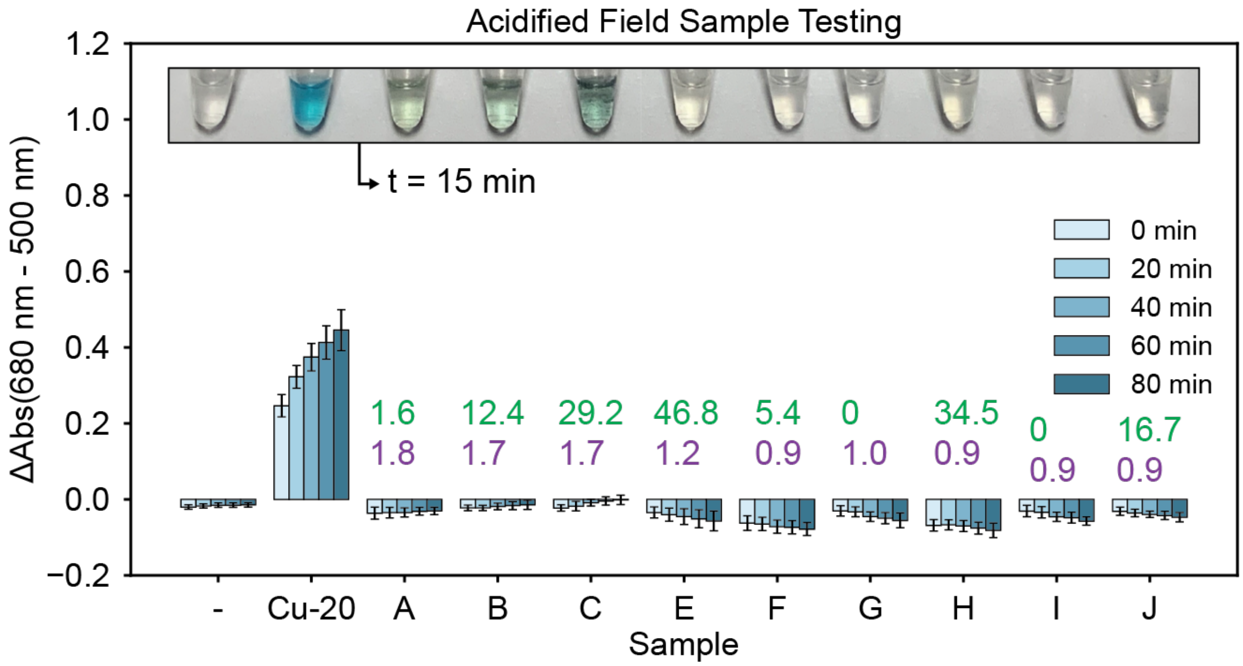

Figure S12: Sensor performance in field samples acidified with nitric acid at 25 °C. "-" indicates rehydration with 18.2 MΩ water, Cu-20 indicates rehydration with 20 µM Cu^{2+} in 18.2 MΩ water, respectively. Field samples are labeled A – J and exclude sample D, which was not acidified prior to analysis. Copper contents of the field samples, measured via FAAS, are listed above the bar plots in µM (top number, green). The pH of each sample, measured with an Orion PerpHecT ROSS Combination pH Micro Electrode, is also listed above the bar plots (bottom number, purple). Upon rehydration, sensor components are 10 mM NaH_2PO_4, 9.4 mM NaOH, 2.5 mM SDS, 0.75 mM TMB, 8 mM H_2O_2, 2.2 M NaCl, and 12.5 µM DTPA. Photographs of sensor tubes are superimposed on each plot at 15 minutes after rehydration at 25 °C. Error bars are ± one standard deviation about the mean of three experimental replicates, each of which contained two technical replicates (n = 6).

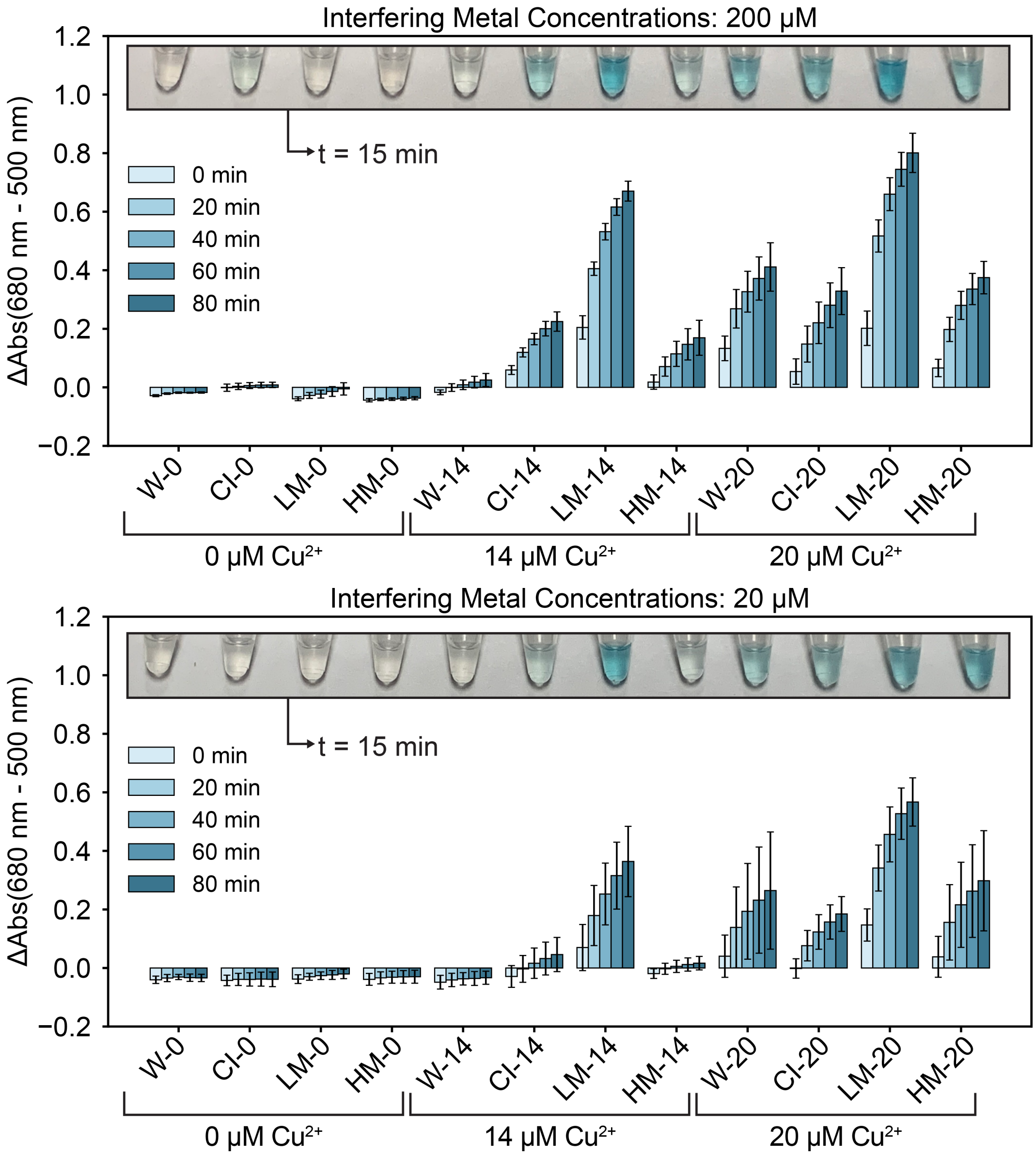

**Category**	**W**: Water	**CI**: Common Ions	**LM**: Light Metals	**HM**: Heavy Metals
**Interfering Metals Added**	None	Zn^{2+}, Mg^{2+}, Ca^{2+}, Fe^{2+}, Fe^{3+}	Co^{2+}, Ni^{2+}, Mn^{2+}, Al^{3+}	Pb^{2+}, Hg^{2+}, Cd^{2+}

Figure S13: Sensor performance in complex solutions at 25 °C. The four categories of interfering metals, denoted W, CI, LM, and HM are described in the table. Interfering metals are each present at the concentrations listed in the plot titles. Cu^{2+} concentration is denoted by "-X", where X is Cu^{2+} concentration in µM. For example, "LM-14" indicates the presence of light metals each at the concentration listed in the plot title and Cu^{2+} present at 14 µM. Upon rehydration with solution, sensor components are 10 mM NaH_2PO_4, 9.4 mM NaOH, 2.5 mM SDS, 0.75 mM TMB, 8 mM H_2O_2, 2.2 M NaCl, and 12.5 µM DTPA. Photographs of sensor tubes are superimposed on each plot at 15 minutes after rehydration at 25 °C. Error bars are ± one standard deviation about the mean of three experimental replicates, each of which contained two technical replicates (n = 6).

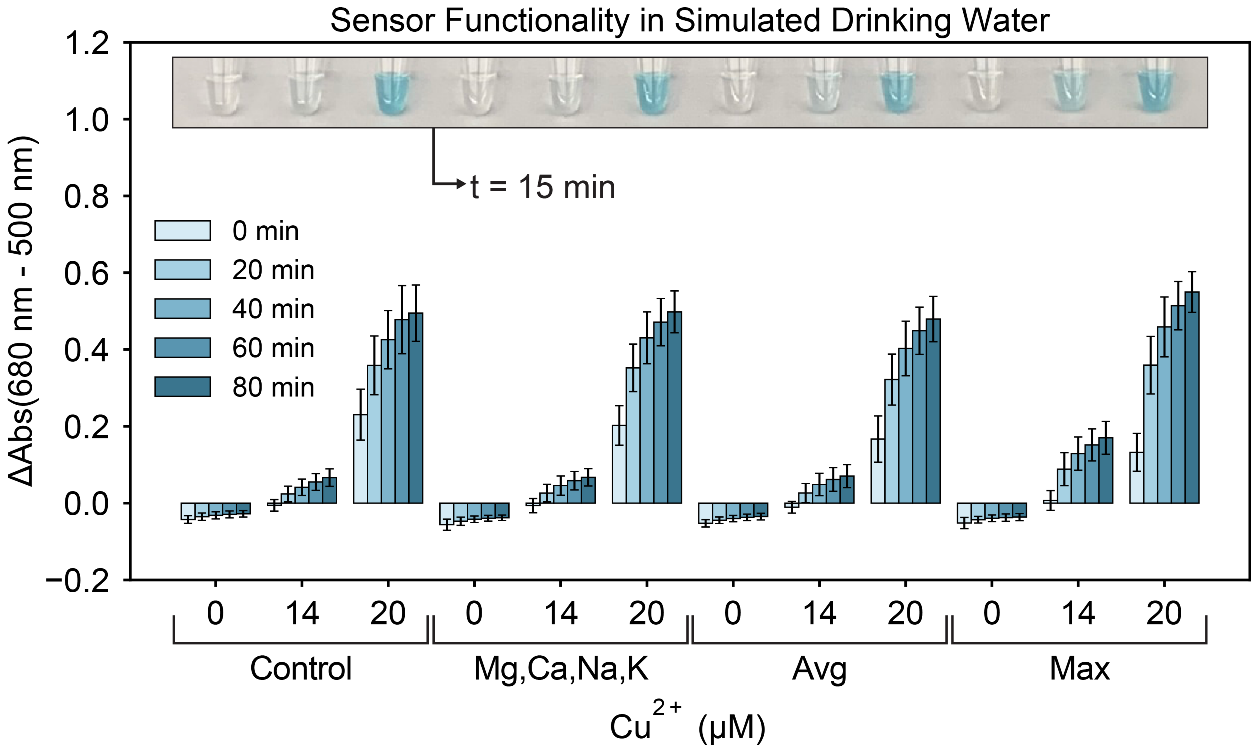

		Control		Mg,Ca,Na,K		Avg		Max	
**Species**	**Salt Used**	**Salt Conc. (μM)**	**Element PPM**	**Salt Conc. (μM)**	**Element PPM**	**Salt Conc. (μM)**	**Element PPM**	**Salt Conc. (μM)**	**Element PPM**
**Ca**	$CaCl_2$	0	0	2500	100	749	30	2500	100
**Cu**	$CuCl_2$	Variable	Variable	Variable	Variable	Variable	Variable	Variable	Variable
**Fe**	$FeCl_3$	0	0	0	0	0.358	0.02	11.6	0.65
**K**	KCl	0	0	5218	204	128	5	5218	204
**Mg**	$MgCl_2$	0	0	1893	46	370	9	1893	46
**Mn**	$MnCl_2$	0	0	0	0	0.0364	0.002	1.8	0.099
**Na**	NaCl	0	0	17010	391	1650	38	17010	391
**P**	NaH_2PO_4	0	0	0	0	3.23	0.1	64.6	2
**Zn**	$ZnSO_4$	0	0	0	0	0.765	0.05	11.5	0.75
**SO_4**	$(NH_4)_2SO_4$	0	0	0	0	2603	250	2600	250
**NO_3**	$NaNO_3$	0	0	0	0	161	10	161	10
**F**	NaF	0	0	0	0	105	2	211	4

Figure S14: Sensor functionality in simulated drinking water at 25 °C. Four categories of interfering ions, denoted Control, Mg,Ca,Na,K, Avg, and Max were studied and are described in the above table. For each category of ions studied, either 0, 14, or 20 μM $CuCl_2$ was added as presented in the plot's x-axis. This drinking water simulation was based on a study by Pehrsson et al[1] and on US EPA Primary and Secondary Drinking Water Standards[2, 3]. Pehrsson et al sampled 144 tap water sources across the United States and determined the concentrations of Ca, Cu, Fe, K, Mg, Mn, Na, P, and Zn. In their report, they provided the mean, median, and maximum concentration of these ions as measured in mg/100 g. In addition to these species, we also studied

some anions as specified in the US EPA Primary and Secondary Drinking Water Standards. The EPA Secondary Drinking Water Standards specify a maximum recommended fluoride concentration of 2 ppm and a maximum recommended sulfate concentration of 250 ppm. In addition, the EPA Primary Drinking Water Standards specify a maximum fluoride concentration of 4 ppm and a maximum nitrate concentration of 10 ppm. The category "Mg,Ca,Na,K" was assembled to contain Mg, Ca, Na, and K at the maximum concentrations measured by Pehrsson *et. al* in their survey. The category "Avg" was assembled to contain Ca, Fe, K, Mg, Mn, Na, P, and Zn at the average concentrations measured by Pehrsson *et. al* in their survey. In addition, the category "Avg" contains 250 ppm sulfate and 2 ppm fluoride to meet the US EPA's Secondary Drinking Water Standards for those species, as well as 10 ppm nitrate to meet the US EPA's Primary Drinking Water Standard for that species. The category "Max" was assembled to contain Ca, Fe, K, Mg, Mn, Na, P, and Zn at the maximum concentrations measured by Pehrsson *et. al* in their survey. In addition, the category "Max" contains 250 ppm sulfate to meet the the US EPA's Secondary Drinking Water Standards for that species, as well as 4 ppm fluoride and 10 ppm nitrate to meet the US EPA's Primary Drinking Water Standards for those species. For this study, lyophilized reactions were rehydrated with the solutions described, plated, and absorbance measured as outlined in the Absorbance Measurement section. Upon rehydration with solution, sensor components are 10 mM NaH_2PO_4, 9.4 mM NaOH, 2.5 mM SDS, 0.75 mM TMB, 8 mM H_2O_2, 2.2 M NaCl, and 12.5 µM DTPA. Photographs of sensor tubes are superimposed on each plot at 15 minutes after rehydration at 25 °C. Error bars are ± one standard deviation about the mean of three experimental replicates, each of which contained two technical replicates (n = 6).

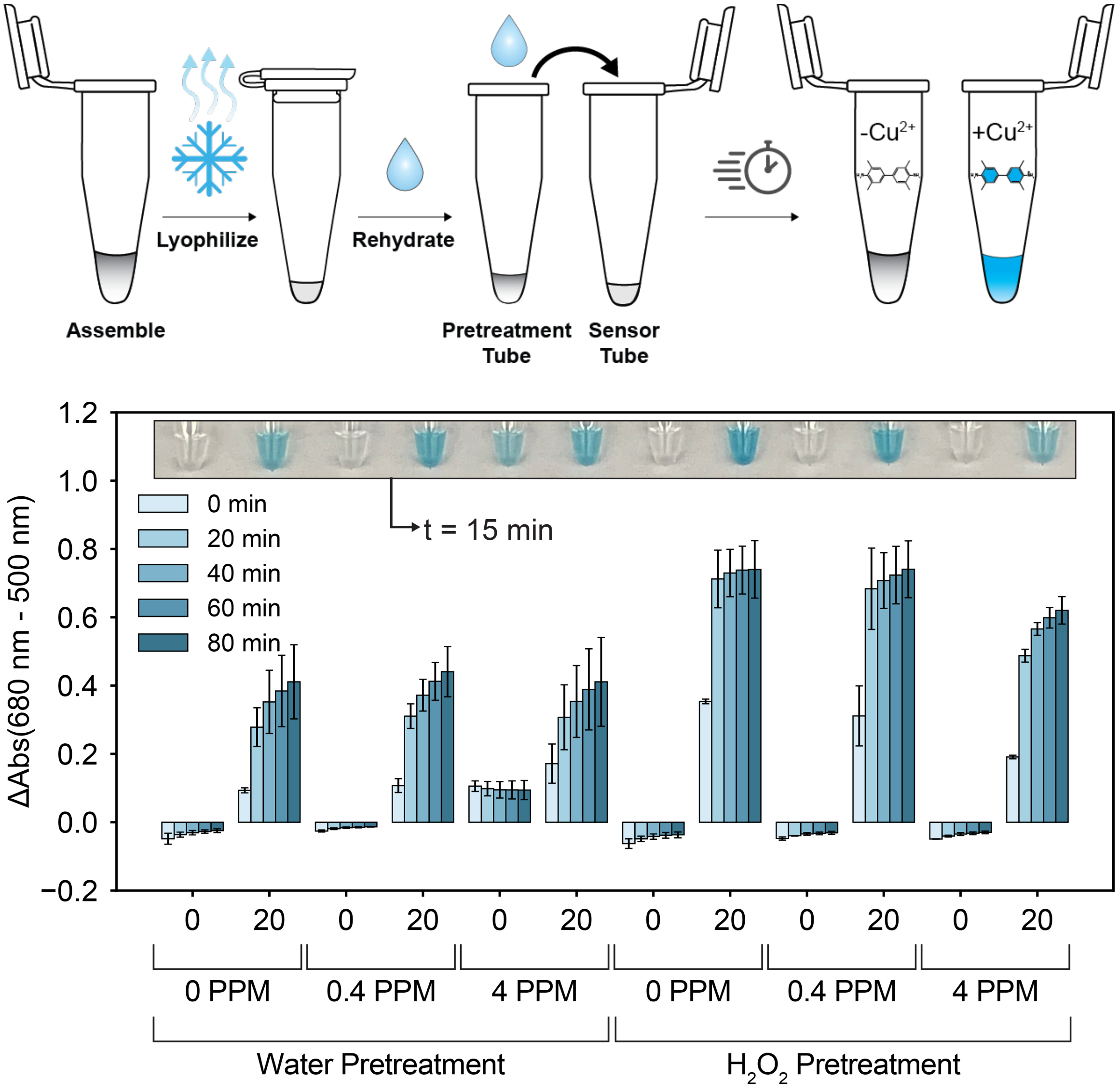

Figure S15: Impact of bleach on sensor performance. To understand the impact of chlorine-based disinfectants on sensor performance, solutions were freshly prepared containing Chlorox germicidal bleach (8.25% sodium hypochlorite) at 0, 0.4, and 4 ppm on a chlorine basis (0.48 g Cl/g sodium hypochlorite) as denoted beneath the plot's x-axis. These solutions contained either 0 or 20 µM $CuCl_2$ as denoted on the plot's x-axis. In the "water pretreatment" condition, 19 µL of solution was mixed with 1 µL of 18.2 MΩ water before being transferred (20 µL) to the sensor tube. Upon rehydration, sensor components are those described previously: 10 mM NaH_2PO_4, 9.4 mM NaOH, 2.5 mM SDS, 0.75 mM TMB, 8 mM H_2O_2, 2.2 M NaCl, and 12.5 µM DTPA. In the "H_2O_2 pretreatment" condition, 19 µL of solution was mixed with 1 µL of 80 mM H_2O_2 before being transferred (20 µL) to the sensor tube. Upon rehydration, sensor components include those described previously along with the addition of 1 mM sodium bisulfite: 10 mM NaH_2PO_4, 9.4 mM NaOH, 2.5 mM SDS, 0.75 mM TMB, 8 mM H_2O_2, 2.2 M NaCl, 1 mM $NaHSO_3$, and 12.5 µM DTPA. Photographs of sensor tubes are superimposed on each plot at 15 minutes after rehydration at 25 °C. Error bars are ± one standard deviation about the mean of three experimental replicates (n = 3).

References

(1) Pehrsson, P.; Patterson, K.; Perry, C. The mineral content of US drinking and municipal water. In Proceedings of the 32nd National Nutrient Databank Conference, Ottawa, ON, Canada, 2008; pp 12-14.
(2) National Primary Drinking Water Regulations | US EPA. 2023. https://www.epa.gov/ground-water-and-drinking-water/national-primary-drinking-water-regulations (accessed 2023 12/05/2023).
(3) Secondary Drinking Water Standards: Guidance for Nuisance Chemicals. 2024. https://www.epa.gov/sdwa/secondary-drinking-water-standards-guidance-nuisance-chemicals (accessed 2024 10/30/2024).
